# Seasonal Mortality and its Impact on Spatial Inequality in Life Expectancy Across Italy

**DOI:** 10.1007/s10680-025-09753-7

**Published:** 2025-10-30

**Authors:** Isabella Marinetti, Dmitri A. Jdanov, Domantas Jasilionis, Marília Nepomuceno, Vladimir Shkolnikov, Fanny Janssen

**Affiliations:** 1 Max Planck Institute for Demographic Research, Rostock, Germany; 2 Population Research Centre, Faculty of Spatial Sciences, University of Groningen, Groningen, The Netherlands; 3 Max Planck—University of Helsinki Center for Social Inequalities in Population Health, Rostock, Germany and Helsinki, Finland; 4 Demographic Research Centre, Vytautas Magnus University, Kaunas, Lithuania; 5 National Research University Higher School of Economics, Moscow, Russia; 6 Netherlands Interdisciplinary Demographic Institute—KNAW/University of Groningen, The Hague, The Netherlands

**Keywords:** Seasonal mortality, Mortality inequality, Regional mortality, Italy

## Abstract

Seasonal mortality fluctuations significantly affect national life expectancy, yet their role in regional inequalities remains underexplored. Understanding this is crucial for targeted health policies aimed at reducing spatial mortality differences. We quantify the impact of seasonal excess mortality on regional life expectancy levels (e_0_) and inequalities in Italy. Using monthly mortality data from the Italian National Statistical Institute by region (N = 20), sex and age between 2005 and 2019, we assessed e_0_ losses due to seasonality by using the general approach of comparing observed e_0_ with minimum achievable levels. Seasonal effects on inequalities in e_0_ were quantified by comparing standard deviations with and without excess seasonal mortality. We then analysed regional contributions using decomposition analysis. Seasonal excess mortality led to an average loss in e_0_ of 1.4 years (1.36–1.70) across Italian regions. This effect was most pronounced in southern and insular regions (Campania and Sicilia), especially for winter-related excess mortality. Our analysis showed that spatial inequality in e_0_ would have been reduced on average by 11.2% by eliminating excess seasonal mortality, mostly due to excess winter mortality (−7.5%). However, during the years with high mortality burdens (2005 and 2015), seasonality contributed to spatial mortality inequality by 10% and 5.2%, respectively. The regional contributions to e_0_ inequality revealed that Campania and Sicilia also had the strongest role in increasing spatial mortality variation throughout the period. The pronounced regional inequalities in e_0_ losses due to seasonal excess mortality contributed significantly to shaping regional e_0_ variation in Italy, mostly due to different impacts of winter mortality within the country

**Supplementary Information:**

The online version contains supplementary material available at 10.1007/s10680-025-09753-7.

## Introduction

Short-term events, such as intra-annual mortality fluctuations due to seasonal variations, can substantially affect annual life expectancy levels (Ballester et al., 2019; Rau, 2007). In fact, seasonal mortality remains a major public health concern, particularly in ageing populations and urban environments (Analitis et al., 2008; Gasparrini et al., 2015). Despite long-term declines in all-cause mortality, the relative contribution of short-term seasonal peaks to annual mortality variation has persisted, or even increased, in certain settings (Healy, 2003; Marinetti et al., 2024). Moreover, the recent research has highlighted how climate change may amplify these effects, increasing the frequency and intensity of temperature-related mortality events (Gallo et al., 2024; Madaniyazi et al., 2021; Vicedo-Cabrera et al., 2018).

These seasonal mortality impacts on life expectancy have been observed to be relatively heterogeneous within Europe (Ballester et al., 2011; Marinetti et al., 2024), varying not only between countries but also across subpopulations (Bennett et al., 2014; Burkart et al., 2014; Hajat & Kosatky, 2009; Lerch & Oris, 2018). Indeed, inequalities in vulnerability (Füssel, 2010; Islam & Winkel, 2017) contribute to diverse patterns of extreme temperature effects on mortality, even within the same country (Kovats & Hajat, 2008; Lerch & Oris, 2018). These spatial differences in vulnerability to seasonal mortality can be driven by several factors, such as socioeconomic status, age structure, comorbidities, and healthcare system preparedness (Füssel, 2010; Islam & Winkel, 2017). Such structural and demographic factors not only shape the baseline health conditions but also the intensity and timing of population responses to seasonal stressors, contributing to within-country heterogeneity in mortality outcomes.

Nevertheless, most research on seasonal mortality has focused on the national level, while the subnational dimension remains understudied, with only some exceptions (Ballester et al., 2011; Martínez-Solanas et al., 2021). These studies found that not only will the impact of temperature-attributable mortality most likely increase, especially in the Mediterranean area, but also its heterogeneity within those countries. Understanding how and to what extent seasonal mortality shapes within-country spatial inequalities in life expectancy is fundamental for targeted health policies and for allocating resources to areas that are majorly affected by these mortality fluctuations. To our knowledge, no previous study has systematically examined how seasonal mortality influences spatial inequality in mortality within countries and how those seasonal regional differences thereby impact mortality levels.

Italy provides an interesting case for investigating these patterns. Firstly, it is among the European countries most affected by heat waves and flu epidemics (Kovats & Kristie, 2006; Michelozzi et al., 2009, 2010). Secondly, the country’s pronounced regional meteorological differentials (Di Giuseppe et al., 2013) and the decentralisation of healthcare to regional authorities (Tediosi et al., 2009) have led to uneven excess mortality patterns across Italian regions during heatwaves, flu epidemics, and cold spells (Conti et al., 2005; Kosatsky, 2005; Meijer et al., 2006; Michelozzi et al., 2016; Rosano et al., 2019). Thirdly, spatial disparities in life expectancy still persist in the country, with life expectancy in the South and North-West lagging behind the North-East and Centre by around 3 years (Italian National Statistical Institute (ISTAT), 2023; Iuzzolino et al., 2011) and healthy life expectancy by around 4 years (Carboni et al., 2024). More recently, Carboni et al. (2024) and Sauerberg et al. (2024) observed a divergence in life expectancy trends between northern and southern Italy, indicating that spatial convergence has stalled.

Several studies have examined territorial mortality differences in Italy, focusing on factors such as education, socio-economic status, and access to healthcare. Across the country, there are marked regional disparities in mortality amenable to healthcare (Lenzi et al., 2013). Socio-economic and geographical inequalities in cancer screening uptake have also been documented (Petrelli et al., 2018). Southern and insular Italian regions have historically exhibited lower healthcare capacity and socio-economic resilience (Graziano & Rizzi, 2016; Ricciardi & Tarricone, 2021; Toth, 2014), which may increase their sensitivity to seasonal stressors such as flu epidemics or heatwaves (Demirtaş, 2023). These studies underscore the need to further explore short-term contributors, such as seasonal mortality shocks, to mortality disparities.

Although it is highly likely that the spatial differences in intra-annual mortality fluctuations in Italy play a significant role to the observed spatial inequalities in life expectancy, comprehensive analyses of the effects of seasonal mortality on overall mortality and life expectancy levels, and how they shape spatial inequalities in Italy, are scarce. A study by Rizzo and colleagues (Rizzo et al., 2007) assessed age-specific patterns of influenza-related deaths between 1969 and 2001, although they did not find any variations in influenza-related mortality when dividing the country into North, Centre, and South regions. Moreover, research on specific high-mortality periods, such as heatwaves, flu epidemics, and extreme cold events, found heterogeneous impacts on mortality within the country (Conti et al., 2005; Michelozzi et al., 2005, 2016).

In this context, the excess mortality approach allows us to quantify the impact of short-term mortality shocks in a comparable way across time and regions (N. Islam et al., 2021; Nepomuceno et al., 2022). A key challenge in analysing seasonal mortality is the definition of the baseline against which excess deaths are measured. Different approaches exist, but here we adopt a counterfactual scenario based on periods of lowest mortality within a year, which provides a consistent benchmark for capturing intra-annual fluctuations, approximating the minimum achievable mortality level and assessing how far each region deviates from it. Moreover, seasonal mortality itself is shaped by multiple factors (e.g. climatic conditions, healthcare preparedness, vaccination coverage, housing quality, and socioeconomic disadvantage) which differ widely across Italian regions. Recognising this interplay is essential for interpreting the contribution of seasonal excess mortality to broader patterns of spatial inequality. 

In this study, we (1) assess the extent to which seasonal excess mortality reduces life expectancy (e_0_) at the regional level in Italy and (2) examine how these effects contribute to spatial inequality in life expectancy in Italy. Particular attention is given to the different effects of specific seasons. Based on existing evidence, we hypothesise that regions in southern and insular Italy were more strongly affected by seasonal mortality shocks and that winter mortality plays a dominant role in shaping regional disparities in life expectancy. Through the decomposition analysis of monthly death counts by region, we integrate the exploration of spatial disparities in mortality, offering new insights into how external shocks shape broader mortality dynamics.

## Data and Methods

### Data

We employed data from the Italian National Statistical Institute (ISTAT) database. We used monthly death counts by 5-year age groups for 20 Italian regions at NUTS2^1^ level and weekly death counts by 5-year age groups for the 5 Italian NUTS1 group of regions (North-East, North-West, Centre, South, Insular). We used yearly population estimates on the 1^st^ of January for each region and group of regions. A map of the regions and groups of regions is included in Supplementary Information, Figure S1. Valle d’Aosta and Molise were not considered in interpreting or analysing the results due to their small population size (around 125 thousand and 303 thousand inhabitants, respectively). All the analyses were carried out separately by Italian region and group of regions, sex, and period (both single-year, 5-year period 2005–2009, 2010–2014, 2015–2019 and all years together). To compute the 5-year and the overall period (2005–2019) analyses, we have aggregated mortality and population counts for the years considered. We decided to stop the analysis before the outbreak of COVID-19 to avoid a misinterpretation of the results due to the unusual mortality fluctuations during the pandemic years.

### Methods

To quantify seasonal mortality impacts on life expectancy and its spatial variation, with a focus on identifying regional and seasonal contributions to inequality, we applied a life table-based excess mortality approach using monthly mortality data. Specifically, to measure the impact of seasonal mortality fluctuations on annual e_0_ by NUTS2 region, we first computed the region-specific annual and seasonal e_0_ employing standard demographic life table techniques (Preston et al., 2001) to the respective age-specific mortality rates. We then computed the *ideal* region-, year-, and sex-specific level of e_0_ using mortality rates of three months with the lowest death counts in each year by region and sex, following Marinetti et al. (2024), and as explained below. Therefore, in contrast to the conventional approach to the study of seasonal mortality, no specific season is employed as the reference level. The baseline is constructed over a period of three months, with no obligation to select consecutive months. This approach facilitates the estimation of the short-term risk factors across diverse seasons, utilising a unified reference level for comparison. The constructed *ideal* e_0_ can be interpreted as the lowest mortality scenario hypothetically achievable in each region each year. This baseline reflects a minimum mortality level within each year, creating a hypothetical scenario where regions are unaffected by major short-term mortality shocks such as flu epidemics, heatwaves, or extreme cold spells. The difference between the *ideal* and the observed (and season-specific) e_0_ is the loss in life expectancy at birth due to excess seasonal mortality.

We decided to focus the analysis on calendar years instead of epidemiological years (common in studies on flu epidemics (Fattore et al., 2024)) because our method identifies the months with the lowest mortality within each year, providing a consistent and flexible baseline that better reflects the timing of seasonal mortality shocks. This approach avoids arbitrary seasonal boundaries and ensures comparability across years and regions (Hajat & Gasparrini, 2016).

#### Mortality Rates in the Absence of Excess Seasonal Mortality (Ideal m_x_)

As explained above, we used the lowest quartile of monthly death counts (i.e. within-year baseline mortality based on 3 months with the lowest death counts across all ages within the year) to estimate the seasonal excess mortality by region. We computed the *ideal* mortality as:

1 $$m_{x,t,r}^{lowest,\;\;month} = \;\frac{{4 \cdot D_{x,t,r}^{lowest,month}}}{{{P_{x,t,r}}}}$$

where ${P_{x,t,r}}$ are the population exposures for the age group *x*, year *t*, and region *r*; $D_{x,t,r}^{lowest,month}$ represents the number of deaths over the 3 months with the lowest death counts for the age group *x*, year *t* and region *r*, multiplied by 4 to reconstruct the whole year as we are basing the analysis only on 3 months. $D_{x,t,r}^{lowest,month}$ is estimated as:

2 $$D_{x,t,r}^{lowest,month} = \mathop {\min }\limits_{M3 \subset \left\{ {1,2, \ldots ,12} \right\};\left| {M3} \right| = 3} \mathop \sum \limits_{month \in M3} D_{x,t,r}^{month}$$

where M3 represents the selected 3 months with the lowest death counts, considering all ages, and $D_{x,t,r}^{month}$ represents the number of deaths in the selected month for the age group *x*, year *t* and region *r*.

#### Correction Factor

Considering death counts by month might overestimate the minimum mortality theoretically achievable in a year. Low-mortality age groups may be susceptible to random events (e.g. traffic accidents), which can lead to elevated rates of mortality. The utilisation of weekly mortality data eliminates the influence of random fluctuations, thereby enhancing the reliability and consistency of the estimates derived. This is attributable to the augmentation of the number of observation points, which increases from three monthly points to thirteen weekly points. Consequently, the post-shock mortality effect (e.g. harvesting effect) is also moderated. Weekly data for NUTS2 regions were not available. Hence, we used the weekly mortality information from the NUTS1 group of regions, dataset to adjust our estimates of *ideal* mortality at NUTS2 level.

To adjust our *ideal* life expectancy estimates, we computed a correction factor based on the information given by the weekly death counts at group of regions level. Therefore, to construct the correction factor we first computed the within-year baseline mortality based on 13 weeks with the lowest mortality within the year (i.e. the lowest quartile of weekly death counts) using the weekly deaths data by group of regions. For each group of regions, year, and sex separately, death counts of 13 weeks with the lowest death counts were summed up to estimate the lowest mortality scenario in a specific year as:

3 $$m_{x,t,g}^{lowest,week} = \;\frac{{D_{x,t,g}^{lowest,week}}}{{{j_t}{P_{x,t,g}}}}$$

where $D_{x,t,g}^{lowest,week}$ represents the sum of the 13 weeks with the lowest death counts for all ages, year *t* and group of regions *g*, considering all ages, and ${P_{x,t,g}}$ the population exposures for age *x*, year *t* and group of regions *g*; *j* is the coefficient to make years with different number of weeks comparable, is equal to ${k_t}/4$ (${k_t} = \frac{{52{*}7}}{365}$ when the year t has 52 weeks, and ${k_t} = \frac{{53{*}7}}{365}$ in case of 53 weeks, according to ISO 8601). Subsequently, using the same weekly data as in (2), we constructed a monthly dataset by groups of regions. We then computed the within-year baseline mortality based on 3 months with the lowest mortality within the year. For each group of regions, year, and sex separately, death counts of 3 months with the lowest death counts were summed up to estimate the lowest mortality scenario in a specific year as showed in Eq. (1).

We calculated the correction factor, *c*, as a ratio between the within-year baseline using weekly and monthly data (based on the same group of regions dataset) for each group of regions:

4 $${c_{x,t,g}} = \;\frac{{m_{x,t,g}^{lowest,week}}}{{m_{x,t,g}^{lowest,month}}}$$

where $$m_{x,t,g}^{lowest,week}$$ and $$m_{x,t,g}^{lowest,month}$$ are computed as in (3) and (1). Therefore, the adjusted *ideal* mortality estimates were computed by multiplying (1) by the correction factor ($${c_{x,t,g}})$$, calculated as in (4):

5 $$m_{x,t,r}^{lowest,\;\;month} = \;\frac{{D_{x,t,r}^{lowest,month}}}{{{P_{x,t,r}}}} \cdot {c_{x,t,g}}$$

where ${c_{x,t,g}}$ is the correction factor that adds the weekly information at group of regions level as explained in (4), and region *r* belongs to group of regions *g* as in Figure S1, Supplementary Information.

#### Spatial Variation

To evaluate the seasonal impact on e_0_ and the spatial variation under various mortality scenarios (eliminating overall seasonal and season-specific excess mortality), we have compared the standard deviations (SD) between Italian regions of the observed e_0_, *ideal* e_0_ and e_0_ in the absence of season-specific excess mortality. To measure the latter, we used as mortality rates:

6 $$m_{x,t}^{ - season} = \;\frac{{D_{x,t}^{ - season}}}{{{P_{x,t}}}}$$

where $D_{x,t}^{ - season}$ are the age- and year-specific death counts, after the elimination of excess deaths of a specific season and *P*_*x*,*t*_ is the population exposures for age *x*, and year *t*. The elimination of season-specific excess deaths was performed by replacing season-specific deaths with those based on death counts in the lowest mortality quartile, as proposed in Marinetti et al. (2024).

#### Decomposition of Seasonal Mortality by Regions

Lastly, to assess the region-specific contribution of seasonality in spatial inequality in life expectancy, we decomposed the difference between observed and *ideal* e_0_ standard deviations by regions, separately by each season, and overall. We applied a modified version of the stepwise decomposition method by age and cause of death (Andreev et al., 2002), using region-specific death counts instead of cause-specific death counts ([sentence eliminated to ensure double-blind review]).

We decomposed the difference between the variation of the observed and *ideal* e_0_ by season and age as follows:

7 $$SD\left( {{e_{0,t}}^{observed}} \right) - SD(e_{0,t}^{upper}) = \Delta SD({e_{0,t}}) = \mathop \sum \limits_{x = 0}^{100 + } \mathop \sum \limits_{region}\Delta SD(e_{x,t}^{region})$$

where $\Delta SD(e_{x,t}^{region})$ are age-, year-, and region-specific contributions of the variations in excess deaths. The analysis was conducted using the statistical software R (version 4.2.3).

## Results

### Life Expectancy Losses Due to Seasonality in Italian Regions

**Fig. 1:**
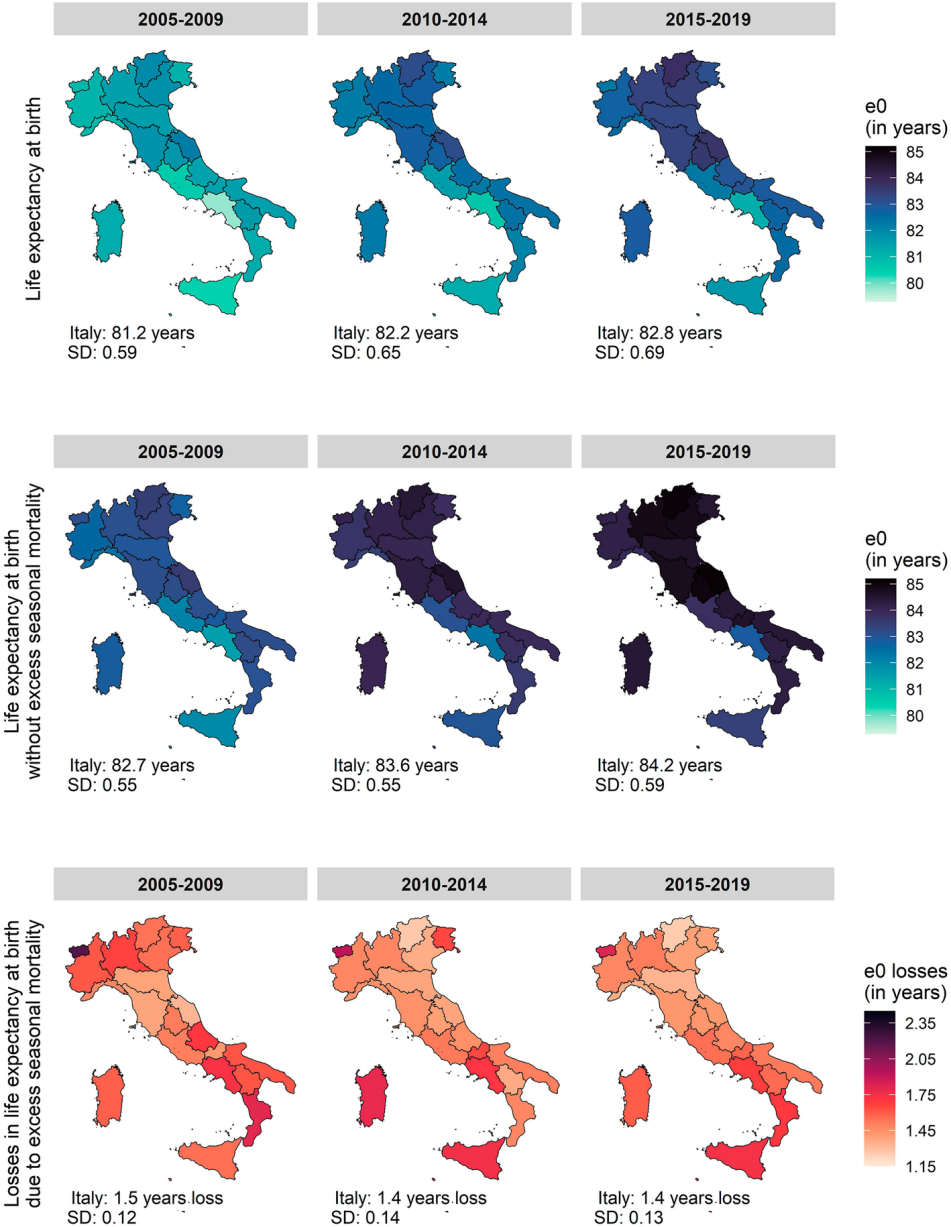
Regional life expectancy at birth, regional life expectancy at birth due to excess seasonal mortality and the related losses, Italian average and the regional standard deviation (SD), Italian regions, total population, 2005–2009, 2010–2014, 2015–2019

Life expectancy at birth (e_0_) in Italy increased from 81.2 years (regional range 79.8–82.2 years) to 82.8 years (regional range 81.2–83.8 years) between 2005 and 2019 (Fig. 1). Life expectancy at birth shows a pronounced spatial gradient, with central and north-eastern regions (such as Marche and Trentino-Alto Adige) having more than a 2-year advantage in life expectancy than the South and Islands (particularly Campania and Sicilia). The maximal average regional differences during 2005–2019 were slightly more pronounced for females (2.7 years) than for males (2.5 years) (Figure S2 and Figure S3, Supplementary Information).

Figure 1 also shows the losses in life expectancy due to excess seasonal mortality, revealing a heterogeneous regional pattern. Overall, we observed the total average of 1.42-year life expectancy loss at the national level and the losses across the regions varying from 1.36 to 1.70 years (1.34–1.74 years for males and 1.35–1.72 years for females) (Fig. 1, Figure S2 and Figure S3, Supplementary Information). The largest losses in e_0_ were experienced in 2005–2009 with a 1.51-year loss (regional range 1.33–1.80 years) at the national level (1.30–1.74-year loss for males, 1.33–1.86-year loss for females). Generally, the highest impact of excess seasonal mortality throughout all the years was observed in insular and southern regions, 1.54–1.64 years for Sardegna, Sicilia, Campania, and Calabria (sex-specific findings in Figure S2, Figure S3 and Table S1 in Supplementary Information; losses across time are presented in Figure S4). When analysing season-specific losses in e_0_ (Figures S2 and S3 in Supplementary Information)_,_ we found that winter excess mortality is the main driver of the overall seasonal excess mortality impact with 2.2-year loss (regional range 1.82–2.66) on average in the analysed period (males: 2.1 (1.70–2.66), females: 2.2 (1.87–2.56), Figures S5, S6, Table S2, Supplementary Information).

### Analysis of Life Expectancy Losses in Extreme Mortality Burden Years

**Fig. 2:**
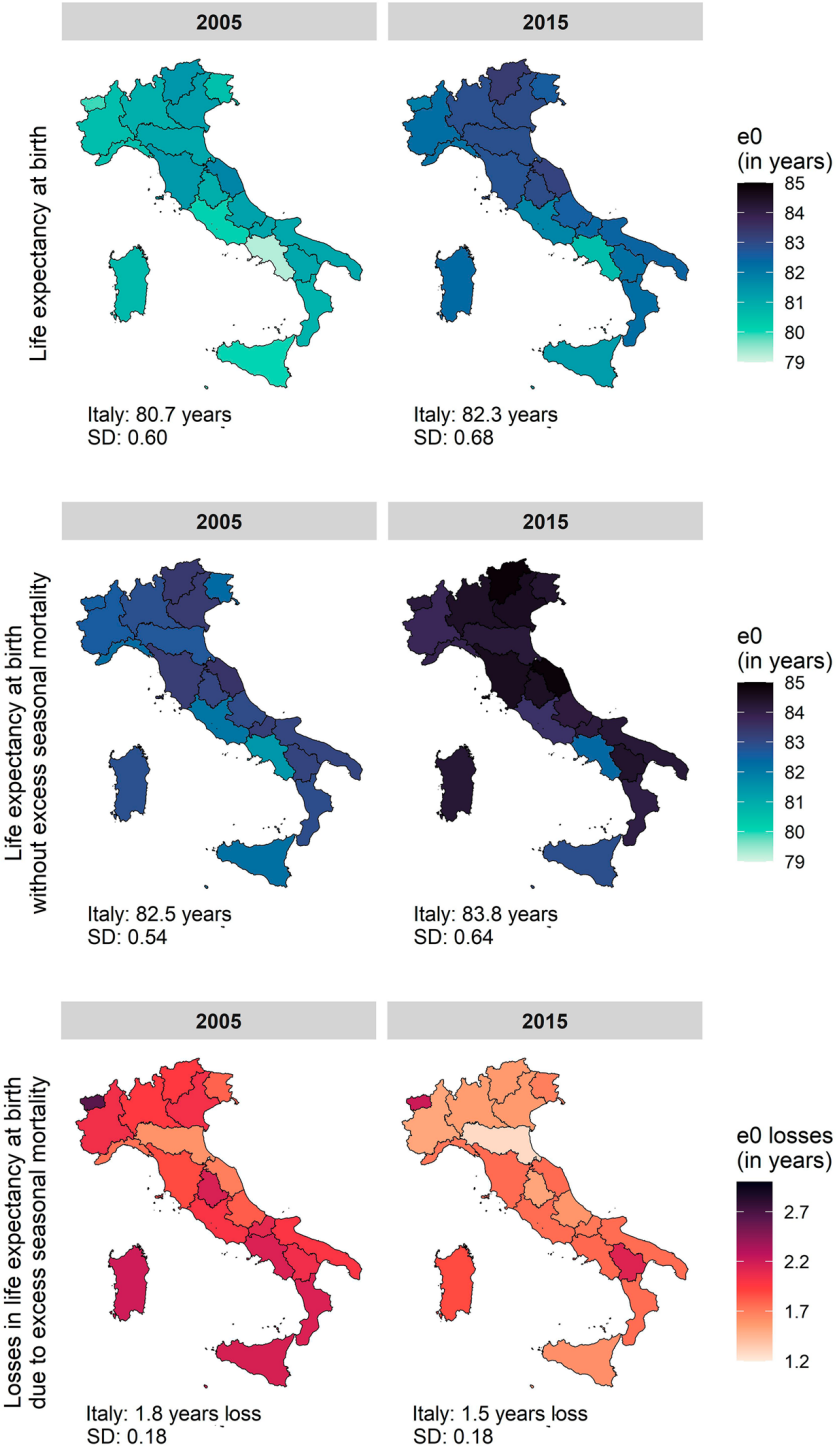
Regional life expectancy at birth, regional life expectancy at birth due to excess seasonal mortality and the related losses, the Italian average and the regional standard deviation (SD), Italian regions, total population, 2005 and 2015

The annual-specific analysis revealed that two years (2005 and 2015) were particularly affected by high mortality losses. The years of 2005 and 2015 were characterised, respectively, by a severe winter, and a strong flu epidemic combined with a heatwave (D’Errico et al., 2020; Michelozzi et al., 2016). Figure 2 shows that the overall life expectancy at birth (e_0_) was 80.7 years (regional range 81.8–79.2 years) in 2005 and 82.3 years (regional range 80.5–83.3 years) in 2015 (Fig. 2). The losses in e_0_ due to excess seasonal mortality were, on average, 1.8 years (regional range 1.6–2.2 years) in 2005 and 1.5 years (regional range 1.3–2.1 years) in 2015. The most significantly affected regions included the Italian islands, especially in 2005 (Sardegna, 2.6-year loss, and Sicilia, 2.15-year loss), southern regions (Basilicata, 2.4-year loss, Campania, 2.3-year loss) and the centre of Italy (Umbria, 2.3-year loss). The sex-specific analysis (Supplementary Information Figures S7 and S8) revealed a similar regional pattern. However, the female population overall was slightly more affected showing 1.9-year loss (regional range: 1.7–2.5 years) in 2005 and 1.5-year loss (regional range: 1.3–2.15 years) in 2015 than the male population (1.7-year loss in 2005, regional range 1.5–2.7 years and 1.4 in 2015, regional range 1.3–2.1 years).

### Spatial inequality in life Expectancy at Birth Due to Seasonality in Italian Regions

As already observed in Figs. 1 and 2, life expectancy inequality in Italy reflects a persistent spatial divide from 2005 to 2019, with north-eastern and central regions generally experiencing lower mortality rates compared to the southern and insular regions. To better understand how seasonal excess mortality affected spatial mortality inequality within the country, we provide a spatial analysis of the life expectancy values observed without seasonal excess mortality and without season-specific excess mortality.

Table 1 shows that removing excess seasonal mortality would have decreased spatial variation on average by 11.2% between 2005 and 2019. These results are mainly attributable to eliminating excess winter mortality (-7.5%), and they most likely correspond to the flu epidemic years. In particular, male life expectancy difference showed a bigger reduction than the corresponding difference among females (respectively, −14.4% and −10.1%), again mostly due to the elimination of excess mortality during winter months (respectively, −9.2% and −6.2%). Generally, male e_0_ showed a higher effect of removing excess seasonal mortality on reducing the regional life expectancy gap. Interestingly, during extreme mortality burden years (2005 and 2015), the elimination of excess seasonal mortality led to varying results. Under this scenario, the spatial difference in e_0_ would decline by 10% in 2005 and only by 5.2% in 2015. This finding suggests that almost all the Italian regions suffered quite similar burdens during 2015, but it was not the case in 2005 (see also Table S3, S4, and S5 in Supplementary Information for the period (2005–2009, 2010–2014, 2015–2019) specific results). To identify the regions that contributed most to this spatial pattern, we further decomposed the differences in e_0_ between observed and *ideal* mortality, as presented in the next section.

**Table 1:** Standard deviations in life expectancy at birth among Italian regions by sex (observed, without overall seasonal excess mortality, without season-specific excess mortality) and percentage difference with the observed standard deviation (%Δ), 2005–2019, 2005 and 2015

SD	2005–2019						2005		2015	
	Total	%Δ	Female	%Δ	Male	%Δ	Total	%Δ	Total	%Δ
Observed	0.64	*–*	0.69	*–*	0.62	*–*	0.60	*–*	0.68	*–*
Without seasonality	0.57	*−11.2%*	0.63	*−10.1%*	0.54	*−14.4%*	0.54	*−10%*	0.64	*−5.2%*
Without winter	0.59	*−7.5%*	0.65	*−6.2%*	0.57	*−9.2%*	0.54	*−10.4%*	0.64	*−5.9%*
Without spring	0.62	*−3.3%*	0.67	*−2.8%*	0.61	*−3.3%*	0.59	*−2.0%*	0.66	*−2.3%*
Without summer	0.65	*1.0%*	0.69	*0.3%*	0.63	*1.3%*	0.60	*0.9%*	0.68	*0.0%*
Without autumn	0.64	*0.5%*	0.70	*1.0%*	0.62	*−0.6%*	0.62	*3.6%*	0.69	*1.4%*

### Regional Contributions to Spatial Inequality in Italy Due to Seasonal Excess Mortality

**Fig. 3:**
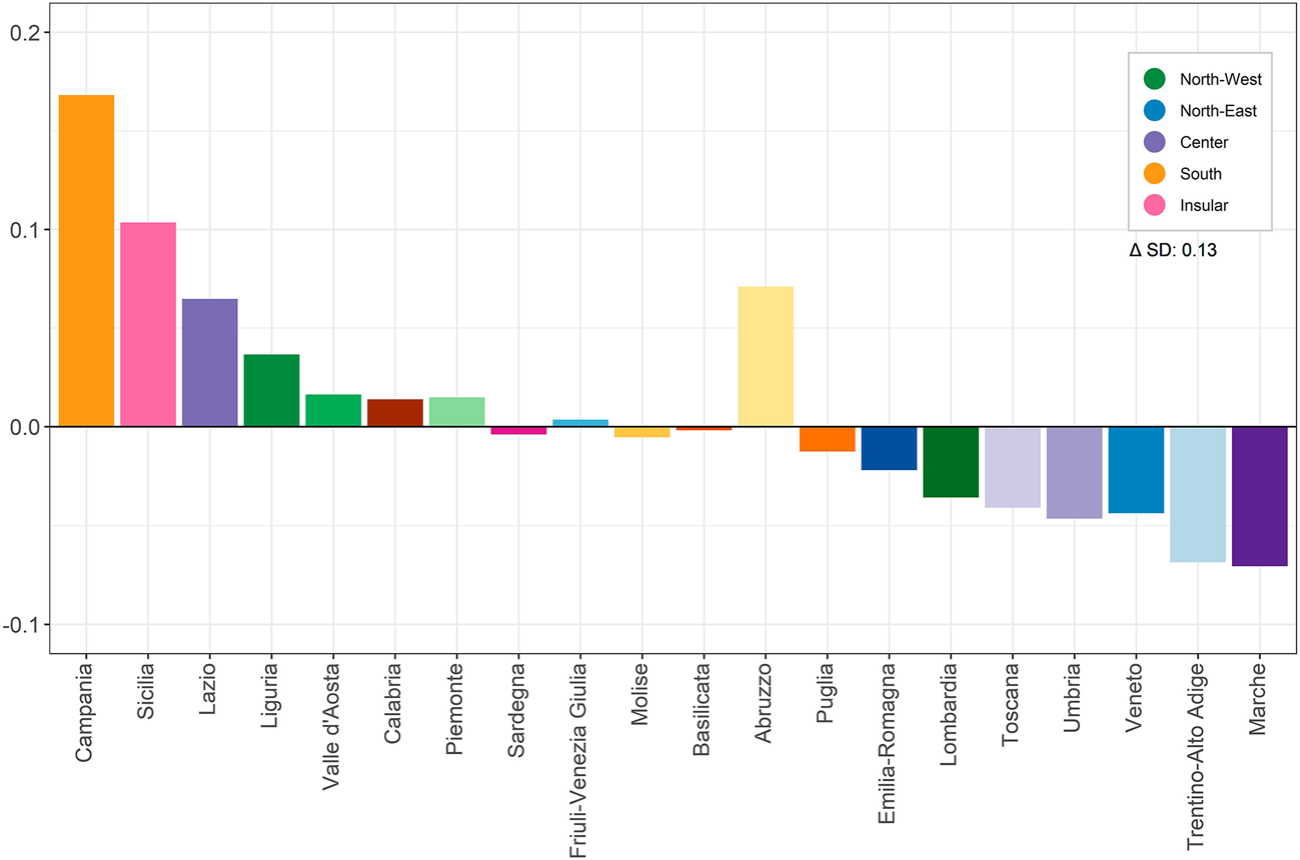
Contributions of Italian regions to the difference in spatial inequality (Δ SD) between e_0_ observed and without excess seasonal mortality, ordered by the observed e_0_ in the region, total population, 2005–2019. *Note*: a positive value means that a specific region contributes to increasing the seasonal impact on life expectancy inequalities, while a negative value reduces it

To assess the contributions of regional mortality rates to national spatial inequality in e_0_, we decomposed the difference in standard deviations between the observed and *ideal* life expectancy (Fig. 3). Figure 3 should be interpreted as follows: a positive value means that a specific region contributes to increasing the seasonal impact on life expectancy, while a negative value reduces it. Moreover, regions are ordered based on the overall observed region-specific life expectancy, from the lowest to the highest. Campania (South) consistently emerged as the region that contributed the most to increasing spatial inequality due to excess seasonal mortality seasonality, with the highest contribution observed in the winter of 2010–2014 and 2015–2019 (0.24, Figure S9 Supplementary Information). One of the two Italian islands, Sicilia, also displayed positive contributions over the whole period, peaking in wintertime 2015–2019 (0.14, Figure S9 Supplementary Information). Conversely, Marche (Centre), Trentino-Alto Adige and Veneto (North-East) consistently contributed to reducing spatial inequality, particularly in 2005 (with a total average of 0.6). The high contribution of the southern region of Abruzzo (yellow bar) reflects the elevated winter excess mortality in the region, despite having the life expectancy at birth higher than the Italian average (see Figure S9, Supplementary Material), together with experiencing an earthquake in April of 2009 that led to an unusual excess of deaths in that region in that year (Alexander, 2011). Analysing the results for each season independently, it is evident that there was a seasonal gradient, with autumn and summer showing the lowest inequality levels in life expectancy (Fig. 9, Supplementary Information). Moreover, we cannot observe significant differences between the female and male populations (Figures S10 and S11, Supplementary Information). These results provide the basis for evaluating the regional and seasonal dimensions of seasonal excess mortality and allow us to explore their contribution to spatial inequality in life expectancy.

## Discussion

### Main Findings

This study assessed the impact of seasonal excess mortality on regional life expectancy and quantified its contribution to spatial inequality across Italy. The findings confirmed our hypothesis that southern and insular regions are disproportionately affected by seasonal mortality and that winter mortality is the main driver of these disparities. In fact, we found systematic spatial variation in the impact of seasonal excess mortality in Italy. Seasonal mortality reduced regional e_0_ levels by an average of 1.4 years (regional range 1.36—1.70) for both males and females in Italy over the years 2005–2019. The largest losses in e_0_ were observed in the South and Islands of the country, especially during the winters of 2005 and 2015. Our analysis showed that spatial inequality in e_0_ would have been reduced on average by 11.2% by eliminating excess seasonal mortality, mostly due to excess winter mortality (−7.5%). Campania (South) and Sicilia (Insular) consistently emerged as the biggest seasonal contributors to the overall spatial inequality in e_0_ throughout the period. These findings highlight not only the persisting spatial disparities in the impact of seasonal excess mortality on e_0_ but also point to the uneven and changing contributions of regions to the seasonal spatial inequality in e_0_ over time.

### Explanation of the Findings

Winter mortality emerged as the main driver of seasonal excess mortality and its spatial inequality, accounting for most of the life expectancy losses across Italian regions. This finding aligns with previous studies, which highlighted the key role of winter-related mortality in shaping life expectancy and mortality levels (Gasparrini et al., 2015; Marinetti et al., 2024) due to the epidemiological processes (cardiovascular and respiratory diseases) that underlie cold-related mortality (Von Klot et al., 2012). Moreover, we identified substantial spatial variation in the distribution of seasonal e_0_ losses in mortality. Specifically, the spatial inequality was driven by the larger impacts on southern and insular regions, which emerged more vulnerable to seasonal excess mortality, particularly during winter. Indeed, while southern regions experience milder winters than the northern part of the country, several factors may explain their higher winter-related mortality burden. These include poorer housing conditions (e.g. inadequate insulation), lower vaccination uptake (La Torre et al., 2010), and limited access to healthcare services (Lenzi et al., 2013; Petrelli et al., 2018). Moreover, while viral spread might usually be homogeneous across regions (Merler et al., 2011), regional differences in the prevalence of comorbidities, healthcare utilisation (Matranga & Maniscalco, 2022), and access to high-quality healthcare (de Belvis et al., 2022) can amplify the health consequences of these exposures. Additionally, lower levels of socioeconomic development and high prevalence of comorbidity may increase sensitivity to seasonal factors. The impact of flu epidemics is therefore mediated not just by exposure but also by adaptive capacity and healthcare preparedness. In fact, the observed regional variation in the impact of seasonal mortality on e_0_ in Italy has the same gradient as healthcare and socioeconomic factors. Southern and insular Italy—where the largest losses in e_0_ due to excess seasonal mortality were observed—has faced significant challenges in healthcare provision. These regions have fewer healthcare facilities, limited access to advanced treatments, and longer waiting times compared to the northern and central regions (Carboni et al., 2024). Such healthcare disparities are further heightened by persistent socioeconomic inequalities, including lower educational and occupational levels in the South and Islands (Lenzi et al., 2013). Consequently, these regions may face challenges in responding effectively to external health stresses, such as seasonal epidemics or extreme temperature events. The inadequate implementation of preventive measures, such as influenza vaccination campaigns, may further reduce population adaptability to seasonal risk factors. Vaccination uptake rates, which are also influenced by socioeconomic status and trust in their efficacy (Bonanni et al., 2015; Giacomelli et al., 2022), may also contribute to these disparities, as lower trust and access can lead to insufficient coverage and reduced population resilience during epidemic seasons. The already existent lower socioeconomic status of individuals in these areas could limit their capacity to respond optimally to health risks, even if healthcare systems were equally well set up across regions. Furthermore, the geographic differences might also be partly driven by disparities in the management of preventive health screening, which is strongly associated with socioeconomic status (Petrelli et al., 2018). These systemic shortcomings have increased mortality rates during periods of severe health stress, such as seasonal epidemics or extreme temperature events, highlighting the interplay between healthcare inequalities and mortality shocks.

We found that, on average, seasonal excess mortality accounts for approximately 11% of the overall spatial variation (measured by the standard deviation) in life expectancy in Italy. This highlights that seasonal dynamics represent a meaningful contributor to regional inequality. Previous research has identified other important drivers of spatial mortality differences, such as smoking and alcohol use (Federico et al., 2013; Grigoriev et al., 2022; Janssen, 2021; Trias-Llimós et al., 2018). These studies highlight the role of long-term behavioural and lifestyle-related risk factors in shaping regional health inequalities. Our study complements this perspective by focusing on short-term fluctuations in mortality, which operate through different mechanisms. Rather than reflecting cumulative behaviours, seasonal excess mortality is closely tied to acute stresses on health systems, climatic exposures, and epidemiological shocks, and therefore provides insight into the resilience and preparedness of populations and healthcare systems. This distinction is important because it shows that regional mortality gaps in Italy are not only the product of long-standing risk factors, but also of the ability to manage and respond to seasonal health challenges.

Analysis of the contribution of seasonality on e_0_ in high-mortality-burden years (2005 and 2015) revealed two distinct patterns. In fact, the year 2005 was characterised by a severe winter, with snowing episodes throughout the whole country (D’Errico et al., 2020), while the flu epidemic of 2015 and the combined effect of a heatwave in the same year(Michelozzi et al., 2016) significantly increased mortality nationwide throughout the year. The larger at-risk population group also aggravated the high burden of mortality in 2015 due to the unusually lower mortality in the summer of 2014 (Michelozzi et al., 2016). Therefore, while in 2005, the high contribution of seasonality on e_0_ (around 10%) was mainly explained by a concentration of winter excess mortality in the first weeks of the year, we observed only halved the contributions in 2015. This result was most likely due to the prolonged flu epidemic season in winter alongside intense heatwave weeks during the summer (Michelozzi et al., 2016), which increased mortality almost homogeneously within the country. The different duration of the high-mortality-burden events of the two years might be the reason for the observed unequal contribution of seasonal excess mortality on e_0_; however, further research is needed to clarify the causal relationship between the length of health stressors and the influence on spatial mortality inequality.

We observed consistent patterns in the impact of seasonal excess mortality on life expectancy for both sexes. However, during specific high-burden years, such as 2005 and 2015, the female population experienced a slightly greater absolute impact from seasonal excess mortality. This difference might be due to sex-specific vulnerabilities to prolonged mortality shocks, potentially driven by differences in healthcare access and use, as well as the higher representation of women in healthcare occupations, which could increase their exposure to health risks. On the other hand, males showed a higher contribution of seasonality on spatial mortality inequalities, suggesting that reducing seasonal excess mortality would lead to a greater decrease in spatial mortality inequality for males compared to females. This effect may be attributed to the fact that excess seasonal mortality among males primarily occurred in regions with a typically lower life expectancy, hence contributing more substantially to spatial inequality. Lastly, although our findings show that winter mortality remains the primary contributor to seasonal life expectancy losses and spatial inequality in Italy during the study period, we also observe a modest but growing contribution from summer mortality (Table S6, Supplementary Information). This aligns with recent mortality projections showing that heat-related mortality is expected to increase due to more frequent and severe heatwaves, ageing populations, and expanding urban vulnerability (Ballester et al., 2023; Brown, 2020; Gallo et al., 2024). While winter mortality is likely to remain highly relevant, especially in socioeconomically disadvantaged regions, these trends suggest that public health strategies must address both winter- and summer-related seasonal risks in future adaptation planning.

### Strengths and Limitations

To our knowledge, this is the first paper that assessed and quantified the impact of seasonal excess mortality across regions in Italy. Integrating the information of monthly mortality data by region and weekly mortality data by group of regions, and analysing these data for the first time, we have obtained a reliable estimation of seasonal and regional excess mortality. Moreover, the data used were provided by the National Statistical Institute (ISTAT), which provides high-quality official statistics based on Eurostat guidelines.

Nevertheless, our study has some limitations. First, two Italian regions, Valle d’Aosta and Molise, have small population sizes, and their estimates could be biased by random fluctuations in mortality. Although the outcomes did not significantly change, we are not considering them in the interpretations and analysis of the results to avoid their misinterpretations.

Second, the overall estimation of the impact of seasonality on e_0_ is a challenging task due to the counterbalancing effects of the different seasons. This is why using intra-annual estimates of mortality offer the important advantages of smoothing out stochastic fluctuations in mortality rates and simultaneously capturing trends and differences between regions. However, the estimates produced depend on the choice of the baseline mortality level to compute the excess deaths estimates. In fact, despite the excess mortality approach being a gold standard for studying short-term risk factors (N. Islam et al., 2021; Mølbak et al., 2015; Nepomuceno et al., 2022), it is directly impacted by the choice of the baseline. Here, we used the method proposed by Shkolnikov et al. (2022) and used in a previous paper (Marinetti et al., 2024). We have also performed a sensitivity analysis on the month used for the construction of the baseline; we found that using the three months is the optimal way to estimate the ideal level of mortality within a year. In fact, as can be observed in Figure S12 (Supplementary Information), for almost every year, region and sex, the months with the lowest mortality levels are June, August and September. Thus, even if we had used these months fixed in the analysis, the results would differ only negligibly. Moreover, using fewer or more months in the computation of the baseline would have led, respectively, to an overestimation or underestimation of the excess deaths estimates. In fact, using only one month would have increased the probability of considering random fluctuations, while using more than 3 months would have masked the actual seasonal fluctuations in mortality within a year (Marinetti et al., 2024). Also using the national average as the ideal mortality level would not have suited the aim of the paper, as it would not have considered region-specific mortality characteristics. Hence, the method proposed was the best choice to overcome stochastic fluctuations of mortality without losing intra-annual mortality information.

Third, it is important to stress the theoretical nature of the baseline scenario, which assumes the absence of any seasonal mortality shocks. While this assumption provides a useful benchmark for estimating the contribution of seasonality, it should be interpreted as a counterfactual rather than a realistic scenario. Nonetheless, our approach offers a consistent framework to quantify seasonal mortality's effects across regions and time because it allows comparable assessments of the regional impact of seasonal mortality shocks.

### Conclusions

Seasonal fluctuations in mortality had substantial effects on both the life expectancy levels and their regional differences, with southern and insular Italian regions emerging as the most affected. These regions face greater challenges due to structural weaknesses in healthcare systems, which leave them less prepared to handle seasonal health crises. Our findings suggest that seasonal mortality is not only a matter of temperature or flu epidemics, but a display of systemic vulnerability, hence the need for regionally targeted public health strategies. In southern and insular regions, this includes improving access to primary care and flu vaccination, strengthening winter preparedness through early warning systems, and investing in energy-efficient housing to reduce cold-related mortality. More broadly, addressing seasonal mortality as a driver of excess mortality and spatial inequality requires integrating climate adaptation into health planning, especially in vulnerable populations where the impact of seasonal shocks is amplified. Furthermore, timely and region-specific data are essential for monitoring health losses linked to seasonality and for informing targeted public health interventions. More evidence and research are needed to understand the consequences of climate change-related risks and to design interventions that reduce the growing threats posed by seasonal mortality.

## Supplementary Information

Below is the link to the electronic supplementary material.

Supplementary file1 (DOCX 3821 KB)

## Data Availability

Monthly death counts and population exposures are openly available on the National Italian Statistical Institute (ISTAT) website (https://www.istat.it/en/), while the weekly death counts were received upon request to ISTAT.

## Footnotes

1 The Nomenclature of Territorial Units for Statistics (NUTS) is a geocode standard developed and used by the European Union for referencing the administrative divisions of countries for statistical purposes. In this hierarchical classification, NUTS1 refers to major territories (groups of regions), while NUTS2 corresponds to regions.
