# Supplementary material for: Seasonal Mortality and its Impact on Spatial Inequality in Life Expectancy Across Italy
Source: Eur J Popul. 2025 Oct 30;41(1):30. doi: 10.1007/s10680-025-09753-7 (PMC12575880; doi:10.1007/s10680-025-09753-7)
Supplement: Supplementary file 1 — Supplementary file1 (DOCX 3821 KB) [file 10680_2025_9753_MOESM1_ESM.docx]

**Supplementary Information – European Journal of Population**

**Title: Seasonal mortality and its impact on spatial inequality in life expectancy across Italy**

**
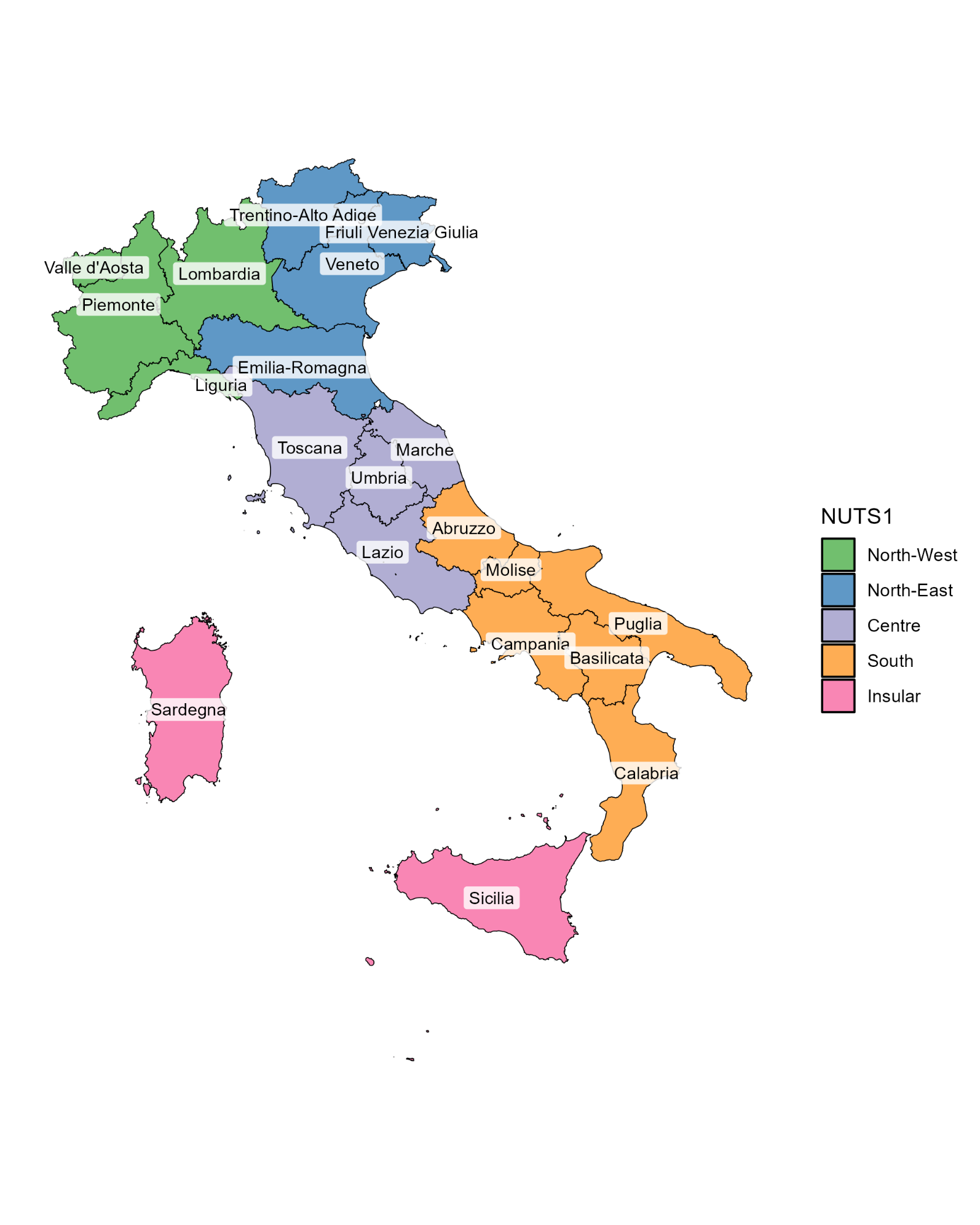
***Figure S1: Italian regions (NUTS2) and groups of regions (NUTS1) used in the analysis*


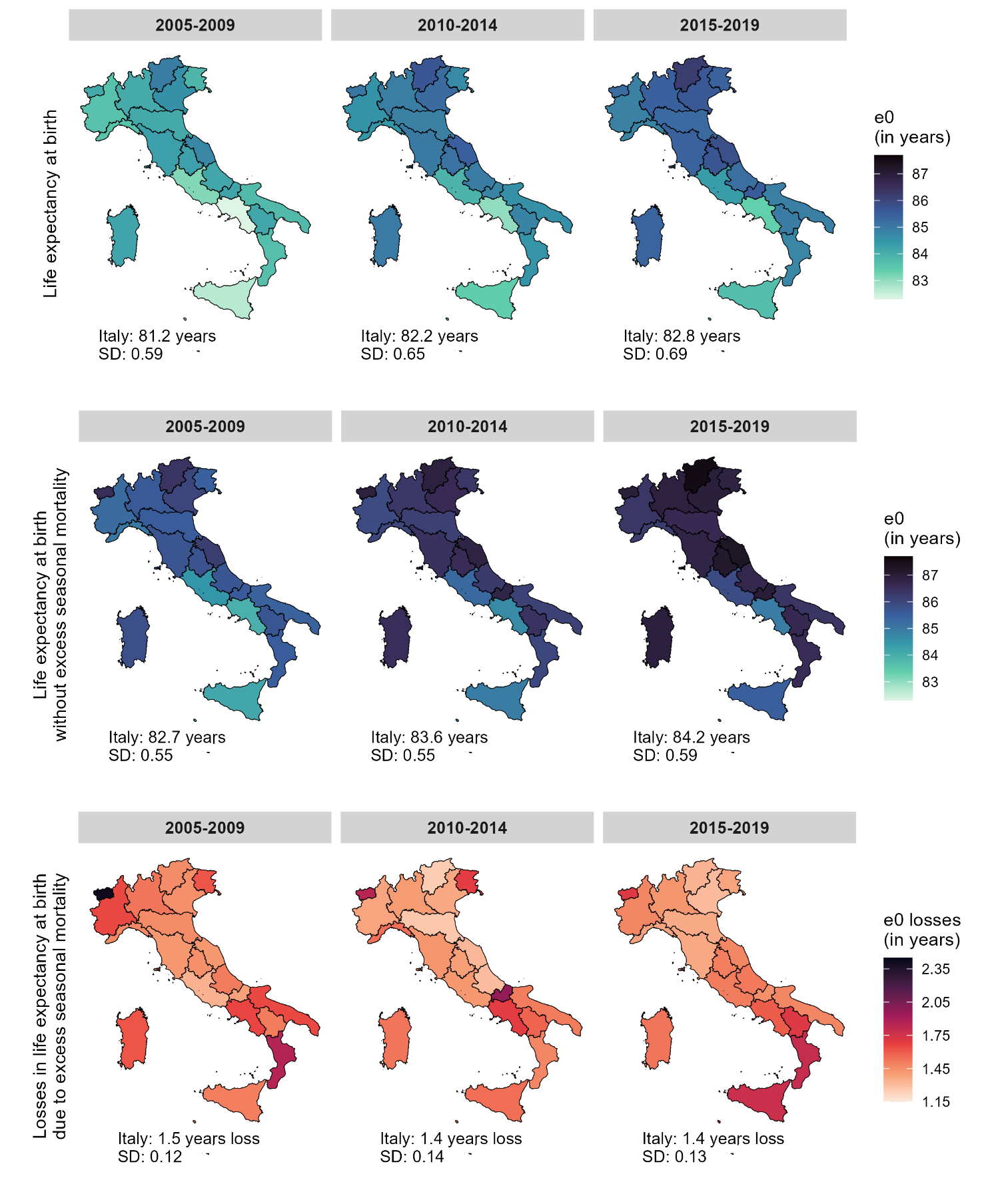
*Figure S2. Regional life expectancy at birth, regional life expectancy at birth due to excess seasonal mortality and the related losses, Italian average and the regional standard deviation (SD), Italian regions, female population, 2005-2009, 2010-2014, 2015-2019*


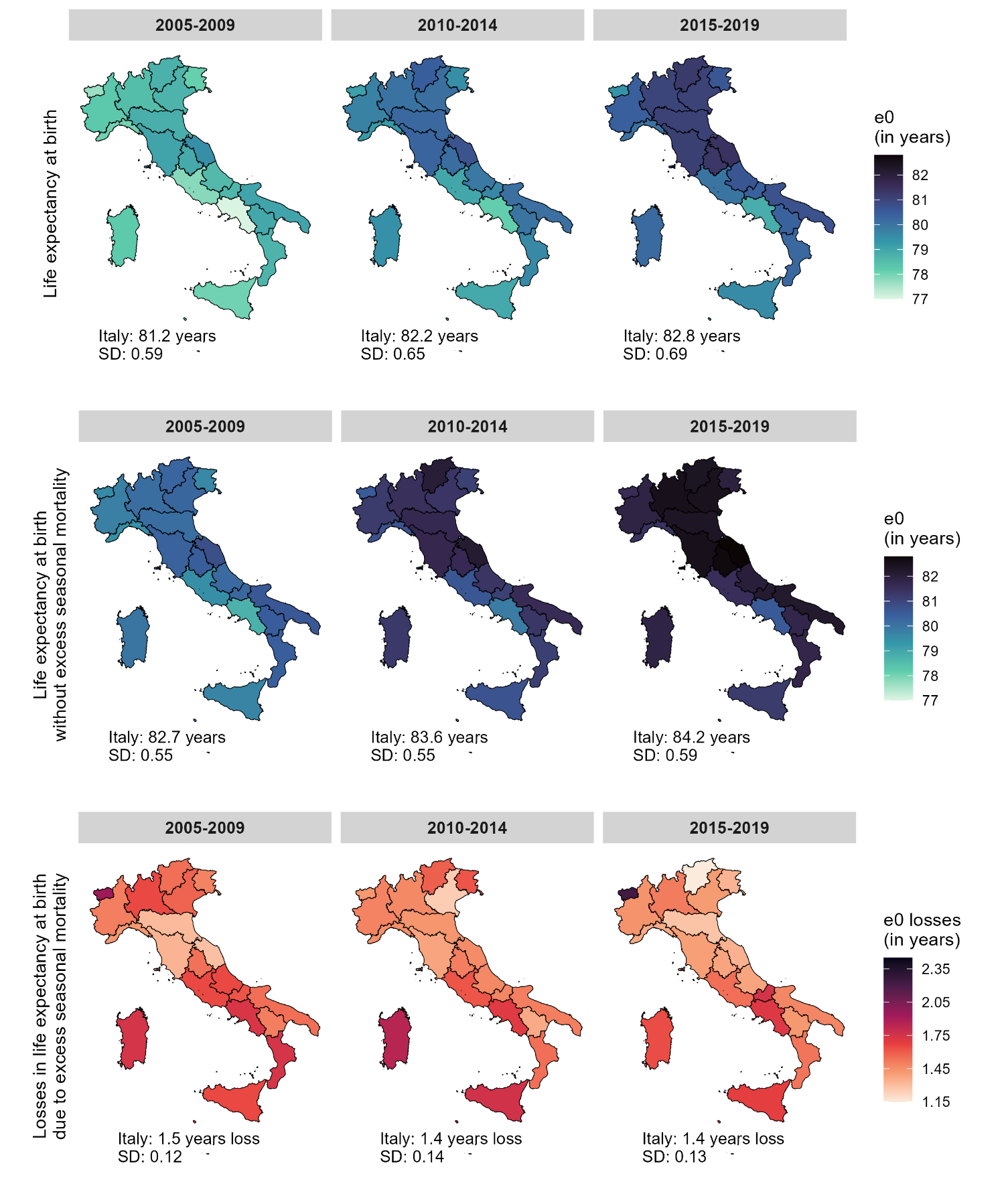
*Figure S3. Regional life expectancy at birth, regional life expectancy at birth due to excess seasonal mortality and the related losses, Italian average and the regional standard deviation (SD), Italian regions, male population, 2005-2009, 2010-2014, 2015-2019*

*Table S1. Losses in life expectancy at birth by Italian regions and sex, 2005-2009, 2010-2014, 2015-2019 and overall period 2005-2019*

| Region | 2005-2009 | | | 2010-2014 | | | 2015-2019 | | | 2005-2019 | | |
| --- | --- | --- | --- | --- | --- | --- | --- | --- | --- | --- | --- | --- |
|  | Total | Males | Females | Total | Males | Females | Total | Males | Females | Total | Males | Females |
| ITALY | 1.52 | 1.50 | 1.47 | 1.41 | 1.37 | 1.38 | 1.44 | 1.40 | 1.42 | 1.42 | 1.38 | 1.39 |
| Piemonte | 1.61 | 1.50 | 1.65 | 1.48 | 1.49 | 1.38 | 1.48 | 1.44 | 1.48 | 1.48 | 1.41 | 1.47 |
| Valle d'Aosta | 2.20 | 1.94 | 2.42 | 1.92 | 1.42 | 1.85 | 1.82 | 2.23 | 1.71 | 1.89 | 1.38 | 1.45 |
| Liguria | 1.48 | 1.42 | 1.47 | 1.45 | 1.45 | 1.55 | 1.36 | 1.36 | 1.38 | 1.34 | 1.48 | 1.43 |
| Lombardia | 1.66 | 1.65 | 1.53 | 1.48 | 1.47 | 1.41 | 1.51 | 1.51 | 1.43 | 1.50 | 1.29 | 1.48 |
| Trentino Alto Adige | 1.54 | 1.55 | 1.46 | 1.27 | 1.59 | 1.25 | 1.26 | 1.15 | 1.33 | 1.32 | 1.35 | 1.30 |
| Friuli-Venezia Giulia | 1.59 | 1.49 | 1.62 | 1.65 | 1.62 | 1.69 | 1.40 | 1.33 | 1.38 | 1.41 | 1.24 | 1.32 |
| Veneto | 1.55 | 1.58 | 1.45 | 1.36 | 1.25 | 1.37 | 1.40 | 1.41 | 1.31 | 1.36 | 1.34 | 1.36 |
| Emilia-Romagna | 1.39 | 1.31 | 1.46 | 1.38 | 1.43 | 1.27 | 1.34 | 1.28 | 1.36 | 1.30 | 1.45 | 1.33 |
| Toscana | 1.38 | 1.33 | 1.42 | 1.45 | 1.38 | 1.43 | 1.43 | 1.40 | 1.37 | 1.38 | 1.58 | 1.36 |
| Umbria | 1.51 | 1.54 | 1.45 | 1.40 | 1.50 | 1.40 | 1.43 | 1.38 | 1.50 | 1.43 | 1.30 | 1.30 |
| Lazio | 1.50 | 1.65 | 1.34 | 1.52 | 1.62 | 1.42 | 1.55 | 1.55 | 1.51 | 1.49 | 1.46 | 1.42 |
| Marche | 1.33 | 1.29 | 1.42 | 1.38 | 1.46 | 1.32 | 1.42 | 1.34 | 1.48 | 1.31 | 1.52 | 1.50 |
| Abruzzo | 1.70 | 1.65 | 1.51 | 1.45 | 1.47 | 1.30 | 1.48 | 1.38 | 1.52 | 1.47 | 1.67 | 1.56 |
| Molise | 1.42 | 1.62 | 1.39 | 1.65 | 1.48 | 1.98 | 1.58 | 1.75 | 1.45 | 1.42 | 1.48 | 1.41 |
| Campania | 1.73 | 1.73 | 1.66 | 1.71 | 1.70 | 1.69 | 1.67 | 1.71 | 1.60 | 1.64 | 1.29 | 1.37 |
| Puglia | 1.62 | 1.55 | 1.65 | 1.50 | 1.50 | 1.51 | 1.52 | 1.48 | 1.48 | 1.47 | 1.57 | 1.61 |
| Basilicata | 1.62 | 1.50 | 1.51 | 1.37 | 1.36 | 1.58 | 1.56 | 1.42 | 1.71 | 1.34 | 1.51 | 1.54 |
| Calabria | 1.80 | 1.74 | 1.86 | 1.48 | 1.55 | 1.48 | 1.70 | 1.53 | 1.80 | 1.60 | 1.68 | 1.47 |
| Sicilia | 1.55 | 1.65 | 1.50 | 1.75 | 1.76 | 1.55 | 1.74 | 1.68 | 1.78 | 1.54 | 1.25 | 1.25 |
| Sardegna | 1.59 | 1.74 | 1.62 | 1.79 | 1.85 | 1.53 | 1.60 | 1.64 | 1.53 | 1.60 | 1.75 | 1.84 |

*
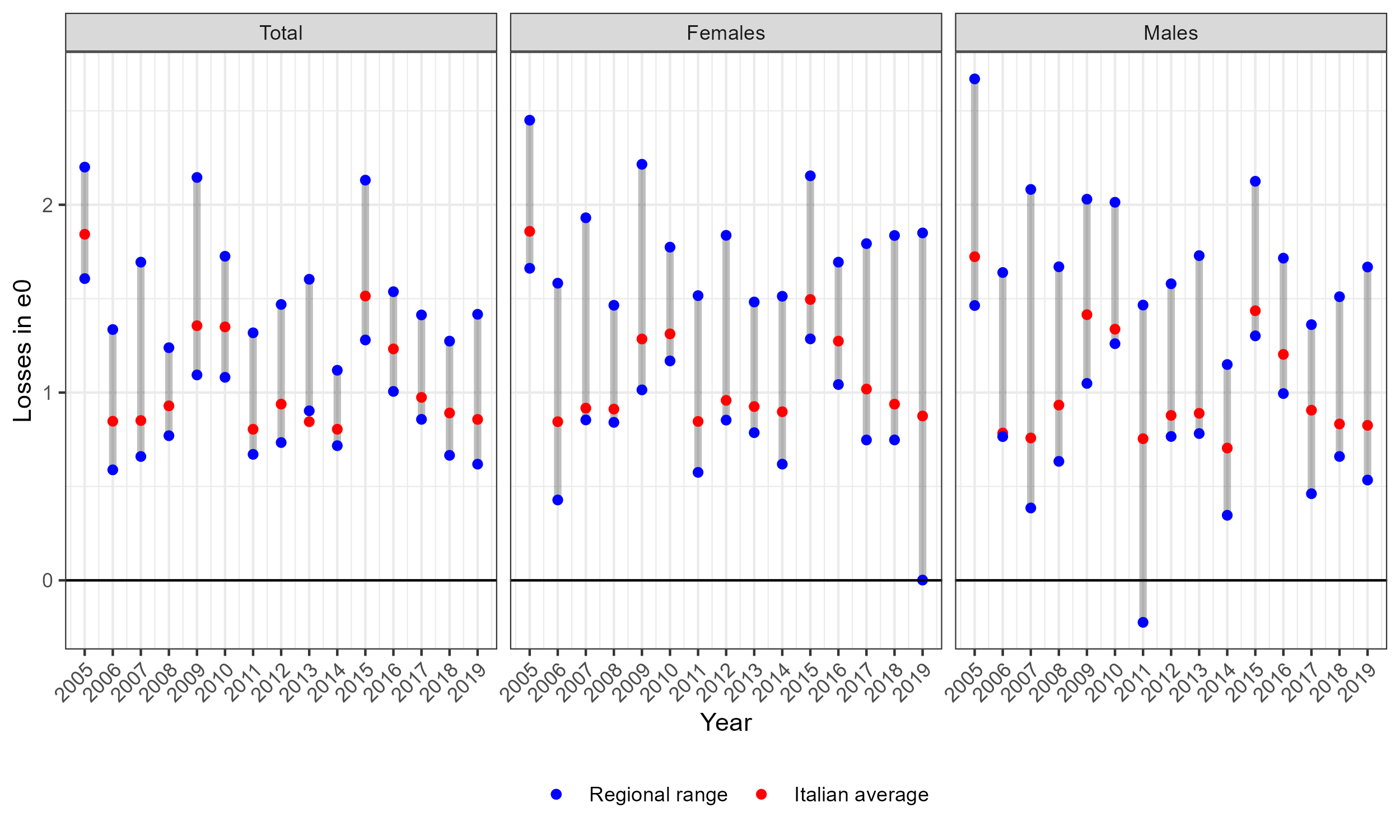
Figure S4. Losses in life expectancy at birth, Italian average and regional range, by sex, 2005-2019*


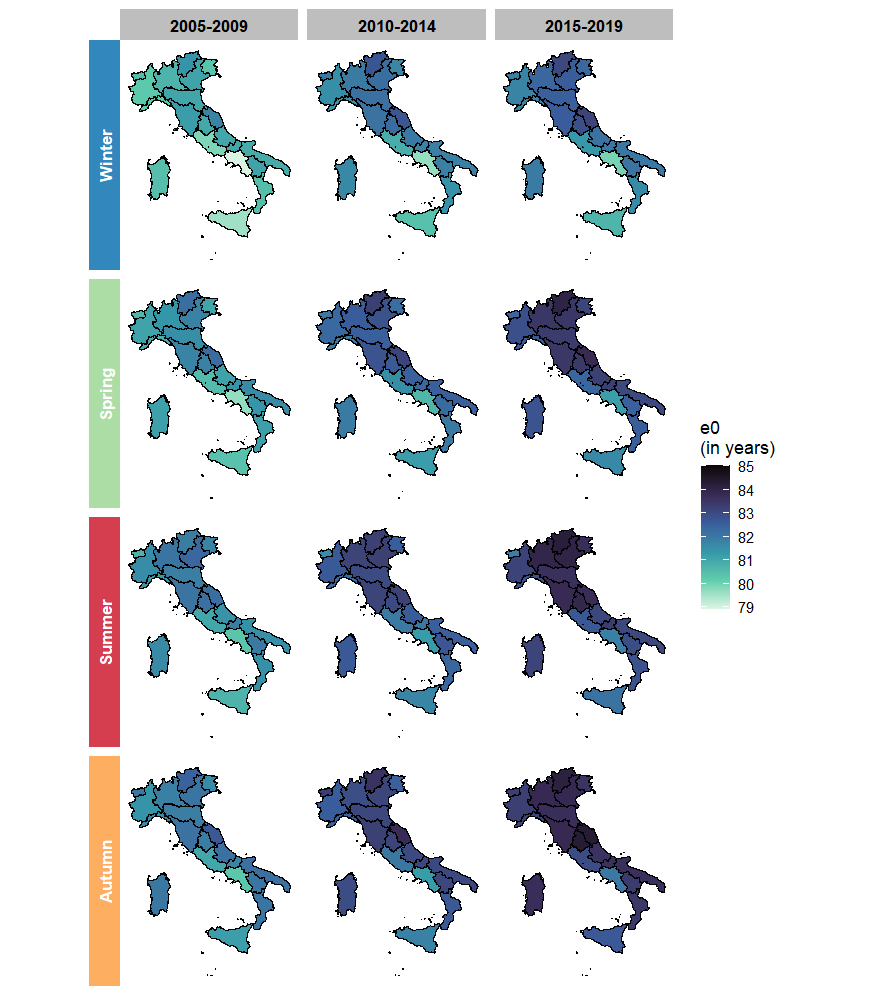
*Figure S5. Life expectancy at birth by season and observed annual estimate, total, population, 2005-2009, 2010-2014, 2015-2019*

*Figure S6. Losses in life expectancy at birth by season, total population, 2005-2009, 2010-2014, 2015-2019*


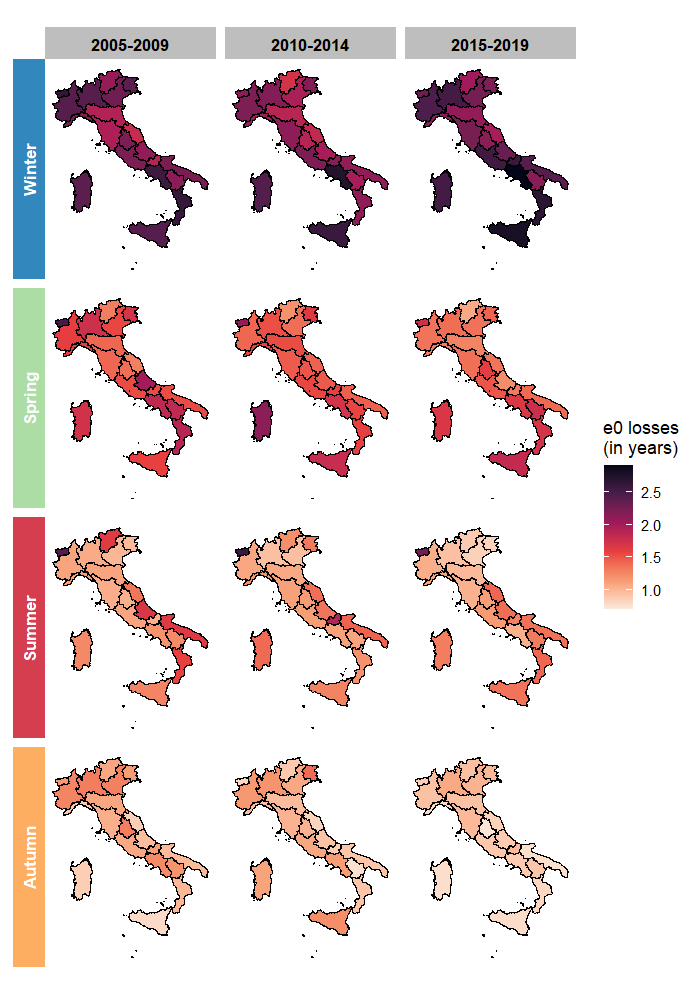


*Table S2. Losses in life expectancy at birth (in years) by Italian regions, by season and sex, 2005-2009, 2010-2014, 2015-2019*

| Region | 2005-2009 | | | 2010-2014 | | | 2015-2019 | | | 2005-2019 | | |
| --- | --- | --- | --- | --- | --- | --- | --- | --- | --- | --- | --- | --- |
|  | Total | Males | Females | Total | Males | Females | Total | Males | Females | Total | Males | Females |
| Winter |  |  |  |  |  |  |  |  |  |  |  |  |
| ITALY | 2.23 | 2.13 | 2.21 | 2.09 | 1.98 | 2.10 | 2.37 | 2.26 | 2.38 | 2.20 | 2.10 | 2.19 |
| Piemonte | 2.40 | 2.57 | 2.13 | 2.21 | 2.13 | 2.15 | 2.45 | 2.43 | 2.38 | 2.31 | 2.16 | 2.35 |
| Valle d'Aosta | 2.62 | 3.24 | 1.94 | 2.12 | 2.50 | 1.18 | 2.30 | 2.28 | 2.60 | 2.27 | 1.83 | 2.51 |
| Liguria | 2.22 | 2.23 | 2.08 | 2.01 | 2.17 | 1.93 | 2.14 | 2.19 | 2.08 | 2.04 | 2.00 | 2.19 |
| Lombardia | 2.40 | 2.28 | 2.35 | 2.18 | 2.15 | 2.09 | 2.51 | 2.45 | 2.43 | 2.32 | 2.23 | 2.28 |
| Trentino Alto Adige | 2.08 | 1.97 | 2.07 | 1.72 | 1.69 | 2.02 | 2.02 | 2.26 | 1.72 | 1.90 | 1.76 | 1.88 |
| Friuli-Venezia Giulia | 2.38 | 2.37 | 2.26 | 2.17 | 2.31 | 2.01 | 2.20 | 2.23 | 2.04 | 2.13 | 1.92 | 2.23 |
| Veneto | 2.27 | 2.23 | 2.20 | 1.96 | 2.06 | 1.73 | 2.25 | 2.12 | 2.25 | 2.09 | 2.01 | 2.06 |
| Emilia-Romagna | 1.92 | 2.07 | 1.72 | 1.88 | 1.89 | 1.81 | 2.09 | 2.16 | 1.94 | 1.89 | 1.73 | 2.00 |
| Toscana | 1.95 | 2.08 | 1.79 | 2.13 | 2.19 | 1.96 | 2.24 | 2.25 | 2.10 | 2.08 | 1.93 | 2.13 |
| Umbria | 2.22 | 2.20 | 2.18 | 1.91 | 1.92 | 1.96 | 2.14 | 2.36 | 1.93 | 2.09 | 2.00 | 2.05 |
| Lazio | 2.21 | 2.01 | 2.37 | 2.22 | 2.05 | 2.36 | 2.53 | 2.44 | 2.54 | 2.30 | 2.41 | 2.12 |
| Marche | 1.80 | 1.97 | 1.65 | 1.84 | 1.83 | 1.85 | 1.99 | 2.18 | 1.78 | 1.82 | 1.70 | 1.89 |
| Abruzzo | 2.10 | 1.83 | 2.10 | 1.98 | 1.87 | 1.94 | 2.32 | 2.47 | 2.10 | 2.06 | 2.00 | 2.05 |
| Molise | 1.90 | 1.84 | 2.10 | 2.16 | 2.35 | 2.08 | 2.59 | 2.45 | 2.74 | 2.09 | 2.22 | 2.11 |
| Campania | 2.55 | 2.47 | 2.51 | 2.72 | 2.70 | 2.67 | 2.90 | 2.79 | 2.92 | 2.66 | 2.67 | 2.57 |
| Puglia | 2.23 | 2.34 | 2.07 | 2.11 | 2.12 | 2.09 | 2.40 | 2.37 | 2.34 | 2.18 | 2.14 | 2.15 |
| Basilicata | 2.14 | 2.06 | 1.98 | 1.99 | 2.35 | 1.81 | 2.15 | 2.50 | 1.82 | 1.93 | 1.75 | 2.08 |
| Calabria | 2.61 | 2.78 | 2.42 | 2.12 | 2.09 | 2.19 | 2.66 | 2.92 | 2.33 | 2.41 | 2.28 | 2.50 |
| Sicilia | 2.41 | 2.41 | 2.45 | 2.58 | 2.50 | 2.44 | 2.78 | 2.92 | 2.60 | 2.46 | 2.31 | 2.54 |
| Sardegna | 2.38 | 2.37 | 2.55 | 2.43 | 2.33 | 2.32 | 2.51 | 2.37 | 2.58 | 2.39 | 2.44 | 2.27 |
|  |  |  |  |  |  |  |  |  |  |  |  |  |
| Spring |  |  |  |  |  |  |  |  |  |  |  |  |
| ITALY | 1.56 | 1.53 | 1.52 | 1.45 | 1.43 | 1.40 | 1.39 | 1.33 | 1.38 | 1.43 | 1.40 | 1.39 |
| Piemonte | 1.59 | 1.68 | 1.44 | 1.42 | 1.29 | 1.45 | 1.35 | 1.34 | 1.32 | 1.41 | 1.34 | 1.40 |
| Valle d'Aosta | 2.50 | 2.57 | 2.35 | 2.08 | 2.12 | 1.47 | 1.75 | 1.58 | 2.21 | 2.02 | 1.89 | 1.94 |
| Liguria | 1.61 | 1.59 | 1.53 | 1.57 | 1.70 | 1.53 | 1.31 | 1.38 | 1.27 | 1.40 | 1.41 | 1.54 |
| Lombardia | 1.75 | 1.60 | 1.76 | 1.51 | 1.40 | 1.54 | 1.38 | 1.36 | 1.33 | 1.51 | 1.49 | 1.43 |
| Trentino Alto Adige | 1.30 | 1.44 | 1.12 | 1.20 | 1.19 | 1.51 | 1.06 | 1.05 | 1.02 | 1.15 | 1.04 | 1.12 |
| Friuli-Venezia Giulia | 1.71 | 1.78 | 1.57 | 1.64 | 1.72 | 1.55 | 1.43 | 1.40 | 1.36 | 1.46 | 1.30 | 1.56 |
| Veneto | 1.56 | 1.38 | 1.64 | 1.38 | 1.41 | 1.26 | 1.36 | 1.31 | 1.33 | 1.35 | 1.36 | 1.29 |
| Emilia-Romagna | 1.42 | 1.51 | 1.30 | 1.53 | 1.32 | 1.68 | 1.27 | 1.35 | 1.14 | 1.33 | 1.28 | 1.36 |
| Toscana | 1.41 | 1.43 | 1.39 | 1.47 | 1.49 | 1.36 | 1.37 | 1.33 | 1.32 | 1.38 | 1.33 | 1.37 |
| Umbria | 1.38 | 1.29 | 1.44 | 1.49 | 1.31 | 1.75 | 1.59 | 1.53 | 1.66 | 1.47 | 1.59 | 1.25 |
| Lazio | 1.53 | 1.39 | 1.67 | 1.56 | 1.44 | 1.69 | 1.49 | 1.41 | 1.52 | 1.50 | 1.61 | 1.35 |
| Marche | 1.29 | 1.23 | 1.38 | 1.43 | 1.45 | 1.44 | 1.35 | 1.38 | 1.30 | 1.30 | 1.31 | 1.25 |
| Abruzzo | 2.00 | 2.02 | 1.77 | 1.47 | 1.37 | 1.45 | 1.22 | 1.24 | 1.16 | 1.49 | 1.42 | 1.52 |
| Molise | 1.54 | 1.28 | 1.93 | 1.58 | 2.18 | 1.17 | 1.33 | 1.15 | 1.53 | 1.35 | 1.45 | 1.43 |
| Campania | 1.84 | 1.86 | 1.74 | 1.72 | 1.70 | 1.70 | 1.66 | 1.63 | 1.67 | 1.67 | 1.67 | 1.64 |
| Puglia | 1.54 | 1.54 | 1.49 | 1.45 | 1.36 | 1.54 | 1.41 | 1.32 | 1.43 | 1.39 | 1.45 | 1.27 |
| Basilicata | 1.84 | 1.76 | 1.68 | 1.56 | 1.56 | 1.73 | 1.77 | 1.94 | 1.60 | 1.53 | 1.52 | 1.51 |
| Calabria | 1.88 | 1.97 | 1.81 | 1.59 | 1.66 | 1.58 | 1.71 | 1.85 | 1.51 | 1.67 | 1.59 | 1.72 |
| Sicilia | 1.58 | 1.51 | 1.70 | 1.79 | 1.55 | 1.82 | 1.82 | 1.86 | 1.75 | 1.59 | 1.57 | 1.57 |
| Sardegna | 1.73 | 1.80 | 1.84 | 2.12 | 1.85 | 2.18 | 1.67 | 1.66 | 1.64 | 1.78 | 1.82 | 1.68 |
|  |  |  |  |  |  |  |  |  |  |  |  |  |
| Summer |  |  |  |  |  |  |  |  |  |  |  |  |
| ITALY | 1.13 | 1.15 | 1.06 | 1.04 | 1.05 | 0.96 | 1.03 | 1.04 | 0.97 | 1.03 | 1.05 | 0.95 |
| Piemonte | 1.10 | 1.06 | 1.07 | 1.08 | 0.97 | 1.11 | 1.04 | 1.05 | 1.01 | 1.03 | 1.00 | 0.99 |
| Valle d'Aosta | 2.42 | 2.59 | 2.24 | 2.58 | 1.89 | 2.61 | 2.27 | 1.83 | 2.95 | 2.33 | 2.49 | 1.93 |
| Liguria | 1.13 | 0.98 | 1.20 | 1.19 | 1.21 | 1.28 | 1.10 | 1.03 | 1.18 | 1.04 | 1.18 | 1.05 |
| Lombardia | 1.05 | 0.92 | 1.10 | 0.94 | 0.80 | 1.03 | 0.94 | 0.81 | 1.03 | 0.93 | 0.99 | 0.81 |
| Trentino Alto Adige | 1.63 | 1.35 | 1.81 | 1.21 | 1.04 | 1.67 | 0.88 | 0.89 | 0.84 | 1.20 | 1.26 | 1.00 |
| Friuli-Venezia Giulia | 0.97 | 1.05 | 0.88 | 1.33 | 1.28 | 1.40 | 0.83 | 0.79 | 0.81 | 0.91 | 0.85 | 0.95 |
| Veneto | 0.99 | 0.89 | 1.04 | 0.95 | 0.82 | 1.00 | 0.85 | 0.75 | 0.90 | 0.85 | 0.92 | 0.74 |
| Emilia-Romagna | 1.09 | 1.00 | 1.16 | 1.07 | 0.90 | 1.20 | 0.98 | 0.87 | 1.04 | 0.98 | 1.04 | 0.88 |
| Toscana | 1.05 | 1.03 | 1.06 | 1.11 | 1.03 | 1.12 | 1.03 | 0.87 | 1.09 | 1.03 | 1.06 | 0.94 |
| Umbria | 1.03 | 0.92 | 1.13 | 1.14 | 1.24 | 1.15 | 1.12 | 1.07 | 1.18 | 1.07 | 1.12 | 0.95 |
| Lazio | 1.09 | 1.01 | 1.17 | 1.13 | 1.04 | 1.23 | 1.15 | 1.11 | 1.15 | 1.09 | 1.16 | 0.99 |
| Marche | 1.33 | 1.58 | 1.15 | 1.37 | 1.14 | 1.61 | 1.42 | 1.45 | 1.36 | 1.31 | 1.30 | 1.29 |
| Abruzzo | 1.68 | 1.29 | 1.81 | 1.34 | 1.16 | 1.42 | 1.38 | 1.46 | 1.25 | 1.39 | 1.46 | 1.27 |
| Molise | 1.03 | 1.32 | 0.97 | 1.91 | 2.14 | 1.82 | 1.28 | 1.28 | 1.37 | 1.29 | 1.30 | 1.49 |
| Campania | 1.19 | 1.08 | 1.23 | 1.11 | 1.05 | 1.15 | 1.03 | 0.96 | 1.07 | 1.04 | 1.11 | 0.93 |
| Puglia | 1.64 | 1.62 | 1.59 | 1.44 | 1.53 | 1.37 | 1.37 | 1.33 | 1.35 | 1.40 | 1.41 | 1.35 |
| Basilicata | 1.24 | 1.09 | 1.17 | 1.10 | 1.31 | 1.09 | 1.32 | 1.12 | 1.50 | 1.05 | 1.13 | 0.95 |
| Calabria | 1.58 | 1.69 | 1.48 | 1.20 | 1.12 | 1.33 | 1.42 | 1.44 | 1.33 | 1.33 | 1.34 | 1.31 |
| Sicilia | 1.26 | 1.21 | 1.38 | 1.27 | 1.10 | 1.24 | 1.35 | 1.35 | 1.35 | 1.15 | 1.13 | 1.15 |
| Sardegna | 1.24 | 1.32 | 1.36 | 1.40 | 0.89 | 1.69 | 1.31 | 1.22 | 1.37 | 1.26 | 1.42 | 1.05 |
|  |  |  |  |  |  |  |  |  |  |  |  |  |
| Autumn |  |  |  |  |  |  |  |  |  |  |  |  |
| ITALY | 1.06 | 1.09 | 0.99 | 0.98 | 0.95 | 0.96 | 0.84 | 0.84 | 0.82 | 0.92 | 0.92 | 0.87 |
| Piemonte | 1.26 | 1.18 | 1.29 | 1.16 | 1.05 | 1.18 | 0.94 | 0.96 | 0.91 | 1.07 | 1.06 | 1.02 |
| Valle d'Aosta | 1.16 | 1.14 | 1.15 | 0.80 | 0.76 | 0.33 | 0.86 | 1.08 | 1.04 | 0.85 | 0.71 | 0.86 |
| Liguria | 0.88 | 0.95 | 0.78 | 0.95 | 1.04 | 0.98 | 0.79 | 0.80 | 0.83 | 0.78 | 0.83 | 0.91 |
| Lombardia | 1.30 | 1.21 | 1.31 | 1.18 | 1.17 | 1.16 | 1.06 | 0.95 | 1.13 | 1.13 | 1.13 | 1.08 |
| Trentino Alto Adige | 1.08 | 0.97 | 1.14 | 0.89 | 1.04 | 1.09 | 0.94 | 0.97 | 0.88 | 0.92 | 0.86 | 0.89 |
| Friuli-Venezia Giulia | 1.14 | 1.11 | 1.13 | 1.39 | 1.36 | 1.45 | 0.98 | 0.94 | 0.96 | 1.02 | 0.97 | 1.04 |
| Veneto | 1.28 | 1.20 | 1.31 | 1.07 | 1.11 | 0.96 | 1.01 | 0.94 | 1.03 | 1.04 | 1.04 | 1.00 |
| Emilia-Romagna | 1.08 | 1.15 | 1.01 | 0.96 | 0.91 | 0.99 | 0.93 | 0.94 | 0.90 | 0.91 | 0.86 | 0.95 |
| Toscana | 1.03 | 1.06 | 1.01 | 1.02 | 0.94 | 1.03 | 0.98 | 0.89 | 1.00 | 0.97 | 0.98 | 0.92 |
| Umbria | 1.31 | 1.28 | 1.33 | 1.01 | 1.05 | 1.10 | 0.73 | 0.90 | 0.62 | 1.00 | 0.99 | 0.96 |
| Lazio | 1.07 | 0.86 | 1.30 | 1.08 | 1.07 | 1.13 | 0.90 | 0.94 | 0.85 | 0.98 | 1.06 | 0.89 |
| Marche | 0.85 | 0.83 | 0.94 | 0.83 | 0.80 | 0.91 | 0.84 | 0.82 | 0.87 | 0.77 | 0.83 | 0.70 |
| Abruzzo | 0.96 | 0.83 | 0.88 | 0.92 | 0.73 | 1.02 | 0.88 | 0.78 | 0.95 | 0.84 | 0.91 | 0.75 |
| Molise | 1.13 | 1.04 | 1.41 | 0.86 | 1.11 | 0.77 | 0.98 | 0.79 | 1.21 | 0.85 | 1.03 | 0.87 |
| Campania | 1.23 | 1.11 | 1.30 | 1.14 | 1.13 | 1.15 | 0.88 | 0.79 | 0.96 | 1.01 | 1.09 | 0.91 |
| Puglia | 0.98 | 0.98 | 0.97 | 0.91 | 0.92 | 0.94 | 0.76 | 0.78 | 0.70 | 0.80 | 0.83 | 0.76 |
| Basilicata | 1.18 | 1.07 | 1.09 | 0.76 | 1.02 | 0.72 | 0.91 | 1.17 | 0.70 | 0.77 | 0.70 | 0.85 |
| Calabria | 0.99 | 0.84 | 1.14 | 0.91 | 0.94 | 0.97 | 0.81 | 0.78 | 0.80 | 0.84 | 0.93 | 0.75 |
| Sicilia | 0.79 | 0.71 | 0.95 | 1.21 | 0.85 | 1.38 | 0.78 | 0.74 | 0.83 | 0.79 | 0.86 | 0.69 |
| Sardegna | 0.86 | 0.88 | 1.05 | 1.10 | 0.90 | 1.14 | 0.77 | 0.73 | 0.80 | 0.85 | 0.93 | 0.75 |

*Figure S7. Regional life expectancy at birth, regional life expectancy at birth due to excess seasonal mortality and the related losses, the Italian average and the regional standard deviation (SD), Italian regions, female population, 2005 and 2015*

*
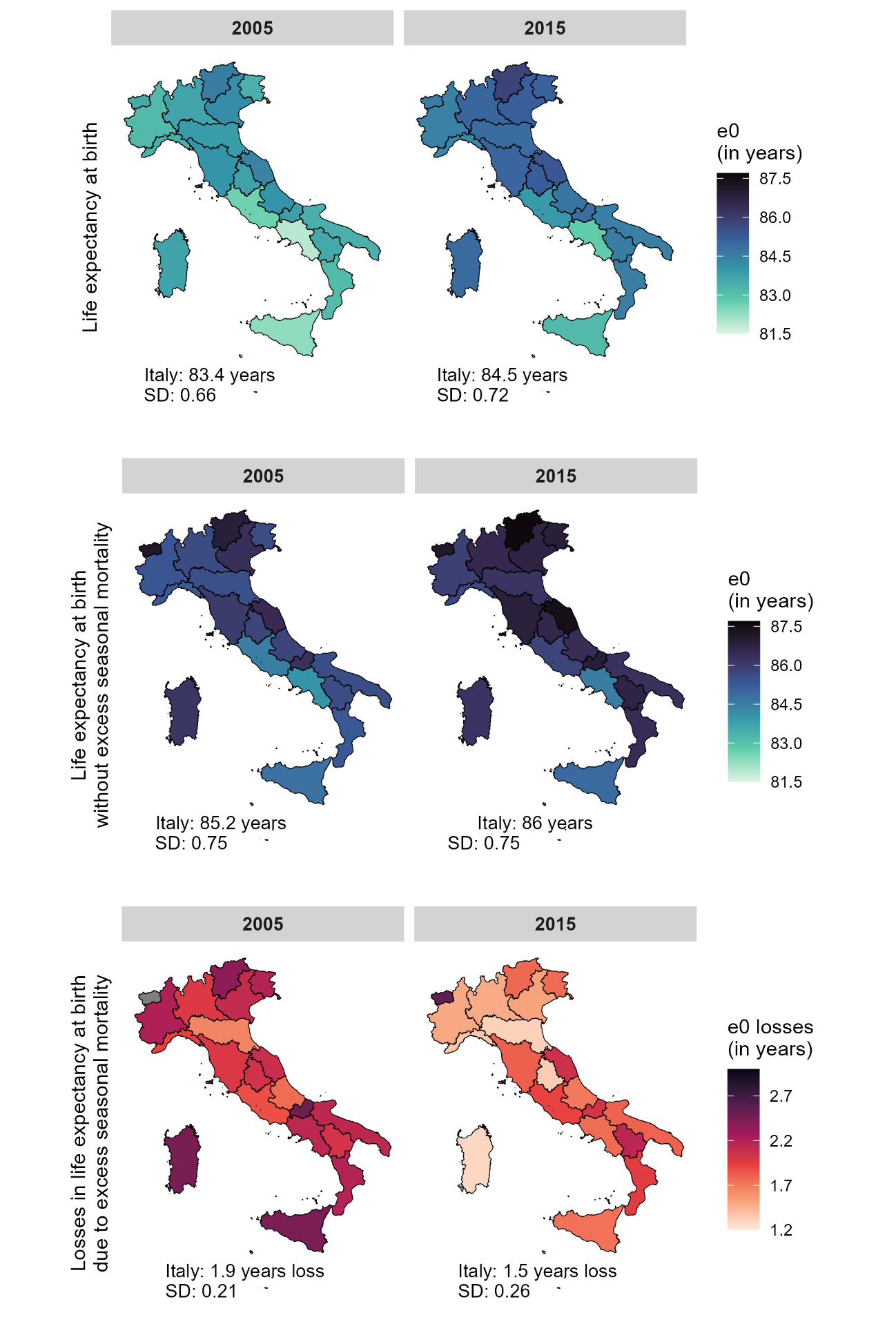
*

*Note: In grey: Aosta Valley, not analysed due to small population size, its values can be considered outliers*

*Figure S8. Regional life expectancy at birth, regional life expectancy at birth due to excess seasonal mortality and the related losses, the Italian average and the regional standard deviation (SD), Italian regions, male population, 2005 and 2015*

*
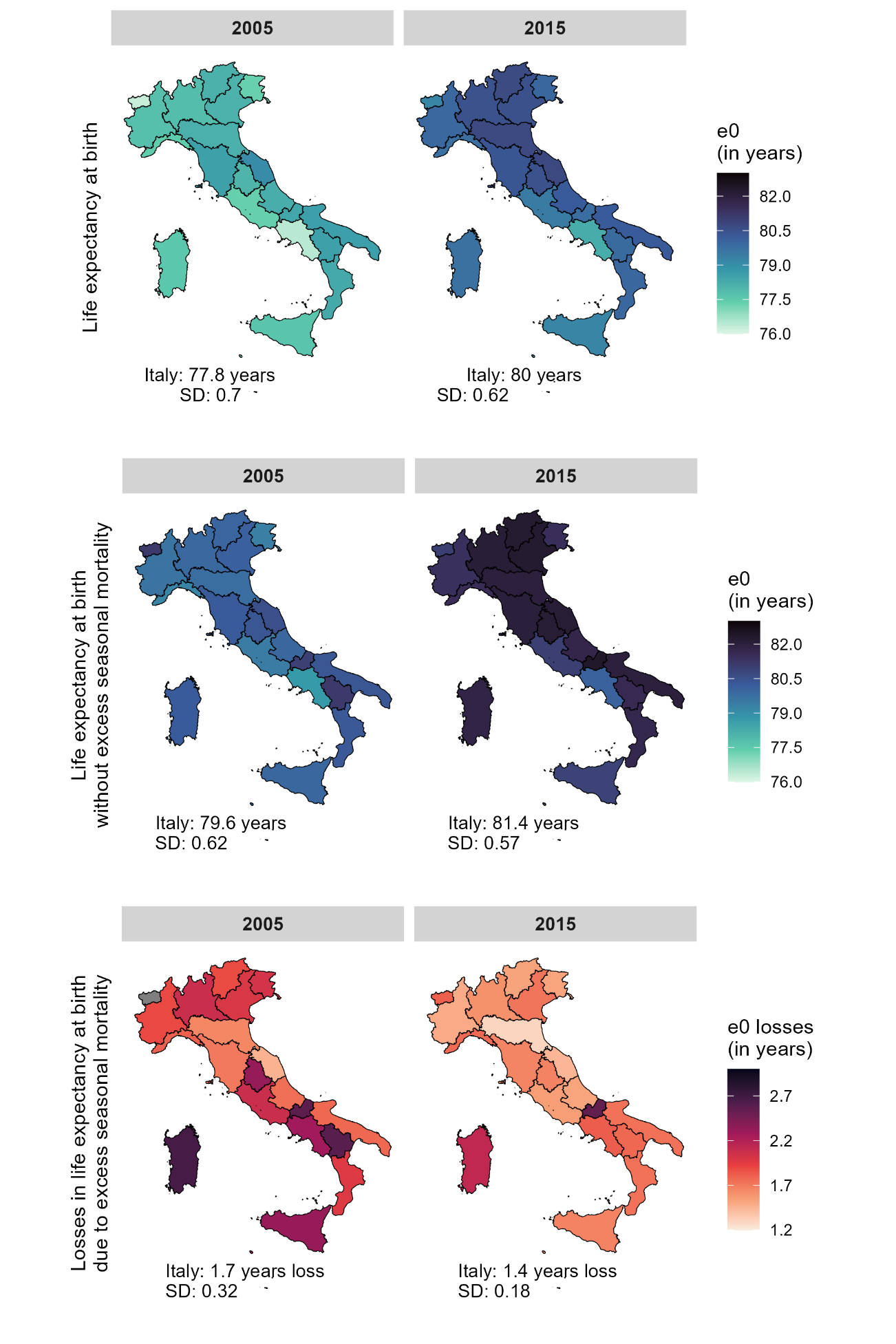
*

*Note: In grey: Aosta Valley, not analysed due to small population size, its values can be considered outliers*

*Table S3. Standard deviations in life expectancy at birth among Italian regions (observed, without overall seasonal excess mortality and season-specific excess mortality), and percentage difference with the observed standard deviation (%*Δ), *2005-2009, 2010-2014, 2015-2019*

| SD | 2005-2009 | %Δ | 2010-2014 | %Δ | 2015-2019 | %Δ |
| --- | --- | --- | --- | --- | --- | --- |
| Observed | 0.59 | *-* | 0.65 | *-* | 0.69 | *-* |
| Without Seasonality | 0.55 | *-7.1%* | 0.55 | *-16.2%* | 0.59 | *-14.5%* |
| Without Winter | 0.56 | *-5%* | 0.59 | *-9.1%* | 0.63 | *-8.6%* |
| Without Spring | 0.58 | *-2.4%* | 0.63 | *-3.4%* | 0.66 | *-4.2%* |
| Without Summer | 0.61 | *3.2%* | 0.65 | *0.8%* | 0.68 | *-1.3%* |
| Without Autumn | 0.60 | *1.7%* | 0.63 | *-1.9%* | 0.69 | *1%* |

*Table S4. Standard deviations in Female life expectancy at birth among Italian regions (observed, without overall seasonal excess mortality and season-specific excess mortality), and percentage difference with the observed standard deviation (%*Δ), *2005-2009, 2010-2014, 2015-2019*

| SD | 2005-2009 | %Δ | 2010-2014 | %Δ | 2015-2019 | %Δ |
| --- | --- | --- | --- | --- | --- | --- |
| Observed | 0.68 | *-* | 0.68 | *-* | 0.71 | *-* |
| Without Seasonality | 0.65 | *-3.8%* | 0.60 | *-11.4%* | 0.63 | *-11.6%* |
| Without Winter | 0.65 | *-4%* | 0.63 | *-7.3%* | 0.67 | *-6.5%* |
| Without Spring | 0.66 | *-1.9%* | 0.67 | *-1.8%* | 0.69 | *-3.8%* |
| Without Summer | 0.69 | *2.2%* | 0.68 | *0.4%* | 0.71 | *-0.9%* |
| Without Autumn | 0.69 | *1.8%* | 0.68 | *0%* | 0.72 | *1.4%* |

*Table S5. Standard deviations in Male life expectancy at birth among Italian regions (observed, without overall seasonal excess mortality and season-specific excess mortality), and percentage difference with the observed standard deviation (%*Δ), *2005-2009, 2010-2014, 2015-2019*

| SD | 2005-2009 | %Δ | 2010-2014 | %Δ | 2015-2019 | %Δ |
| --- | --- | --- | --- | --- | --- | --- |
| Observed | 0.57 | *-* | 0.63 | *-* | 0.67 | *-* |
| Without Seasonality | 0.51 | *-11.7%* | 0.56 | *-13%* | 0.57 | *-16.2%* |
| Without Winter | 0.53 | *-7.6%* | 0.58 | *-8.3%* | 0.61 | *-10.2%* |
| Without Spring | 0.55 | *-2.5%* | 0.62 | *-2.4%* | 0.65 | *-4.1%* |
| Without Summer | 0.58 | *2.7%* | 0.65 | *2.2%* | 0.67 | *-1.1%* |
| Without Autumn | 0.57 | *-0.3%* | 0.62 | *-2.1%* | 0.68 | *0.3%* |


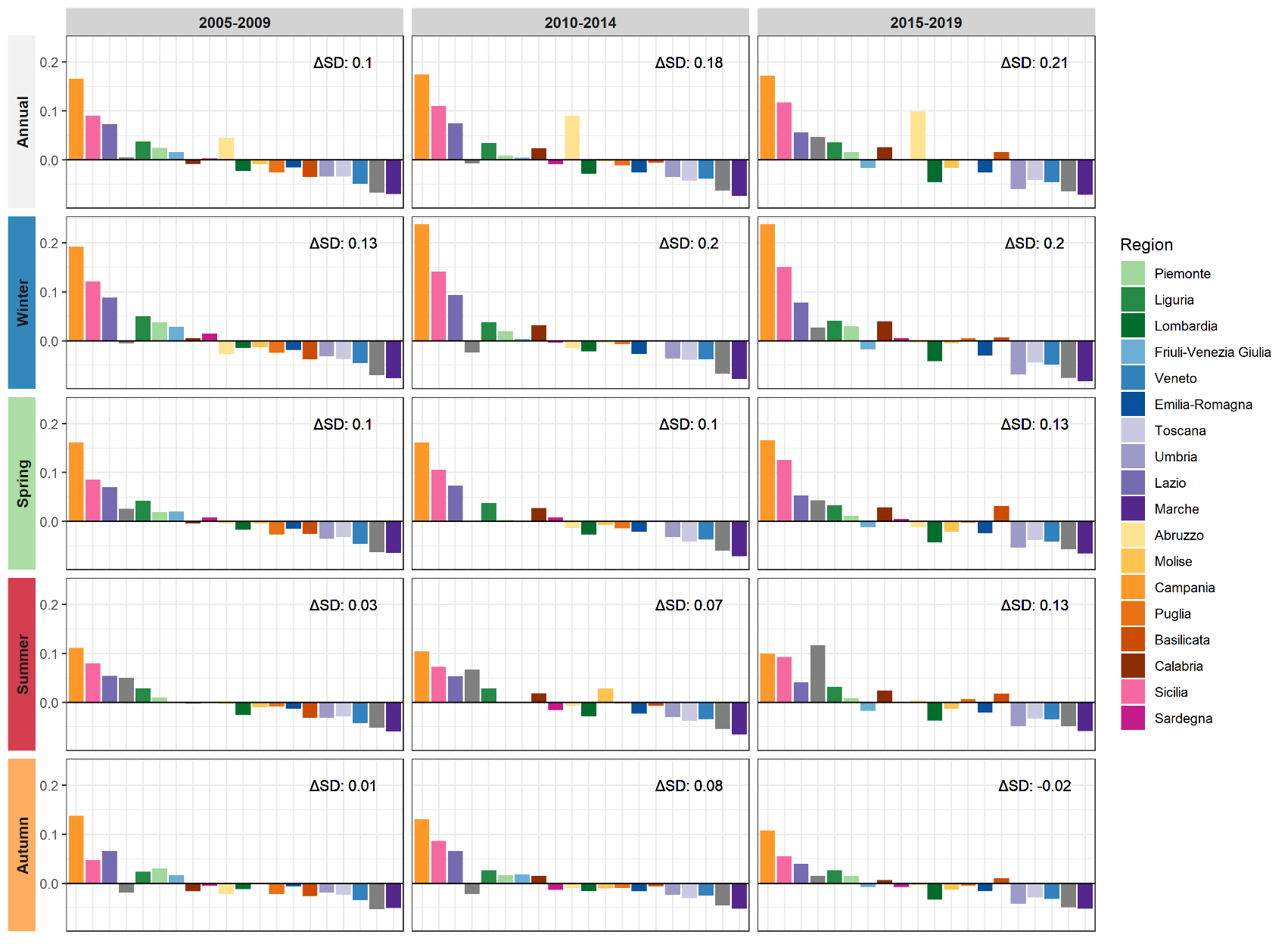
*Figure S9. Contributions of Italian regions to the difference in spatial inequality (Δ SD) between e0 observed and without excess seasonal mortality, analysed independently by each season, ordered by the observed e0 in the region, total population, 2005-2009, 2010-2014, 2015-2019*

*
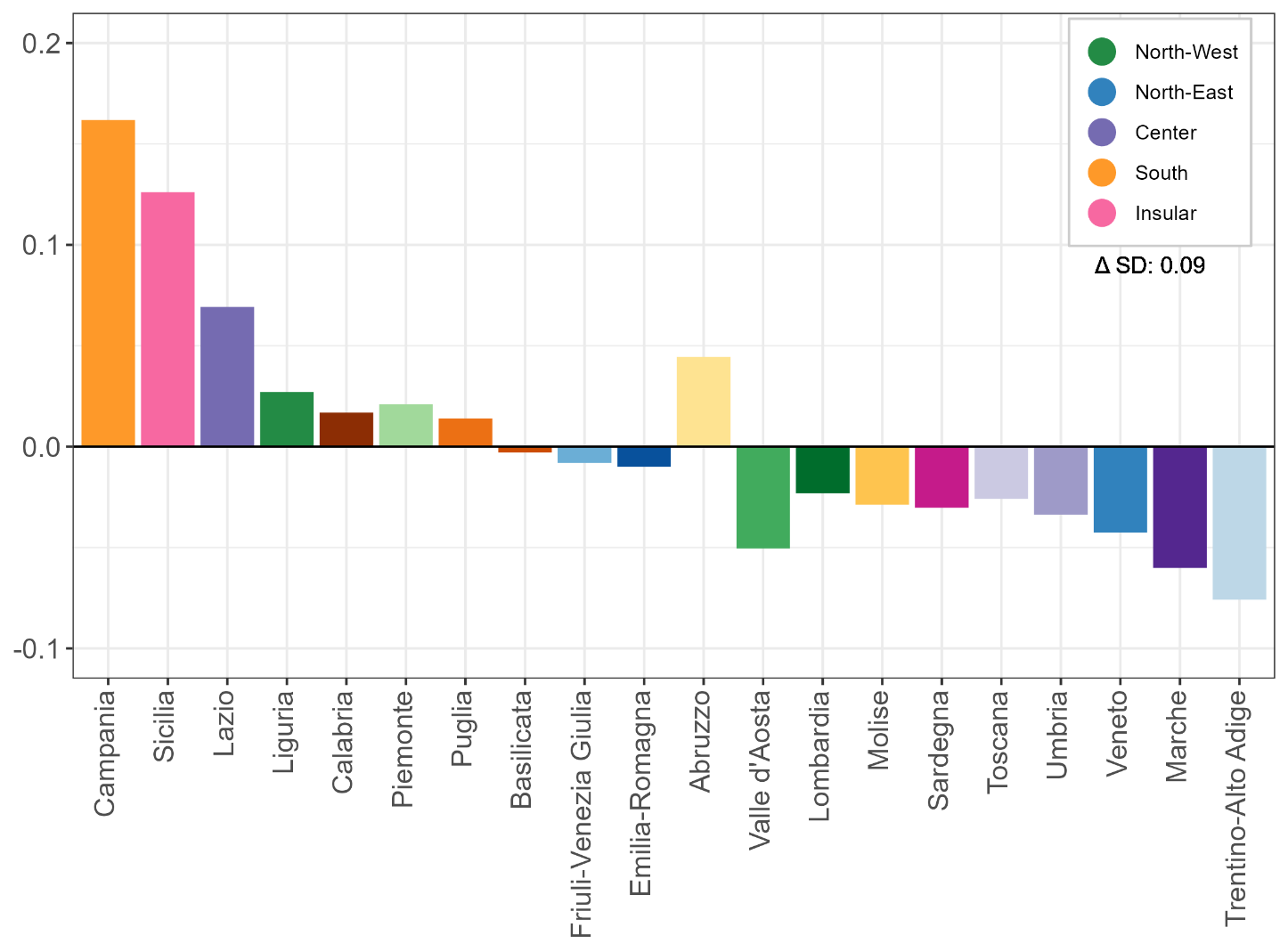
Figure S10. Contributions of Italian regions to the difference in spatial inequality (Δ SD) between e0 observed and without excess seasonal mortality, analysed independently by each season, ordered by the observed e0 in the region, female population, 2005-2019*

*
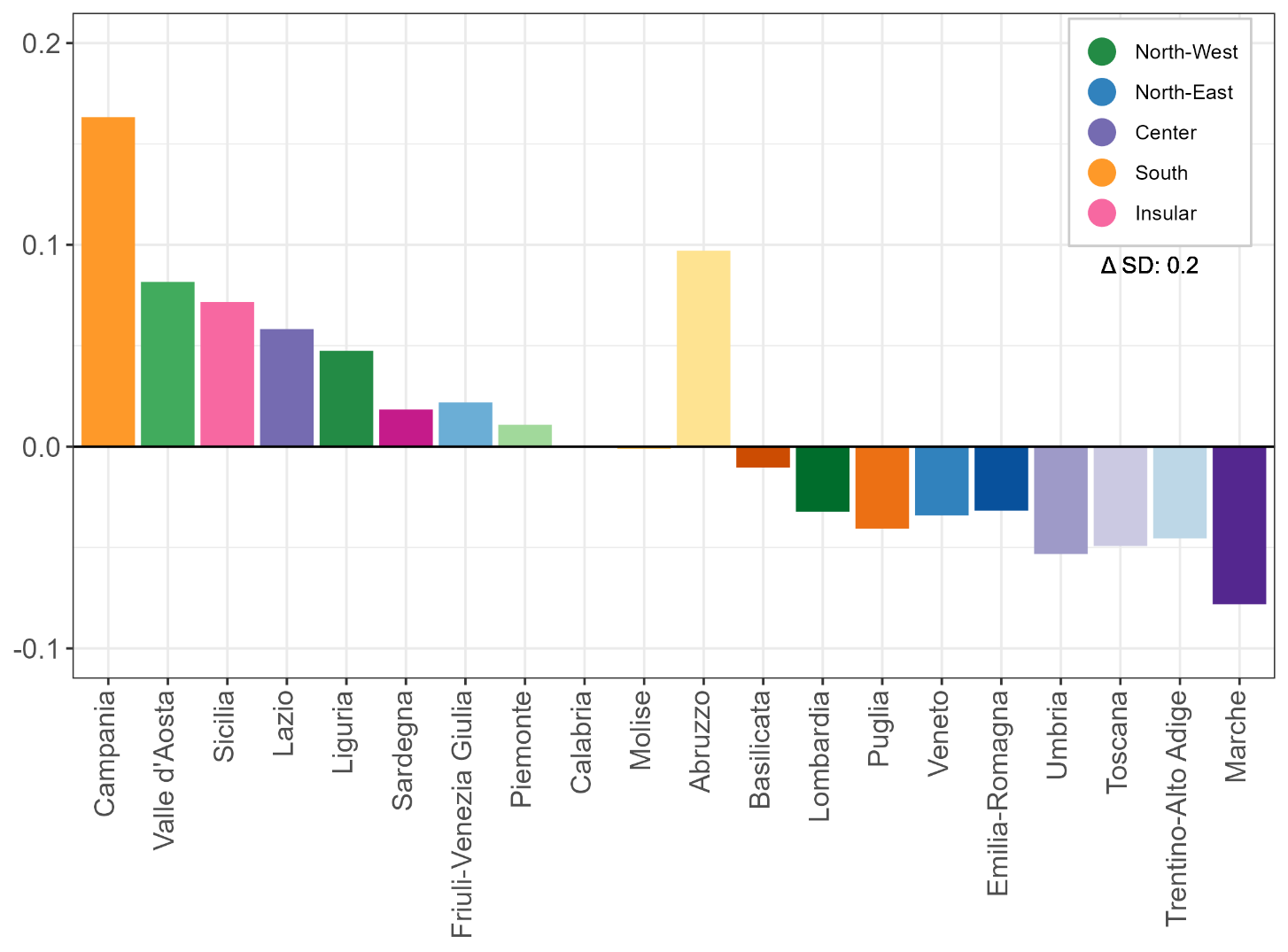
Figure S11. Contributions of Italian regions to the difference in spatial inequality (Δ SD) between e0 observed and without excess seasonal mortality, analysed independently by each season, ordered by the observed e0 in the region, male population, 2005-2019*

*Table S6. Contributions of Italian regions to the difference in spatial inequality (Δ SD) between e0 observed and without excess seasonal mortality, annual and by season, by sex, 2005-2009, 2010-2014, 2015-2019 and 2005-2019*

| Region | 2005-2009 | | | 2010-2014 | | | 2015-2019 | | | 2005-2019 | | | |
| --- | --- | --- | --- | --- | --- | --- | --- | --- | --- | --- | --- | --- | --- |
|  | Total | Males | Females | Total | Males | Females | Total | Males | Females | Total | Males | Females |  |
| Annual |  |  |  |  |  |  |  |  |  |  |  |  |  |
| ITALY | 0.1 | 0.15 | -0.04 | 0.18 | 0.12 | 0.07 | 0.21 | 0.28 | 0.16 | 0.13 | 0.2 | 0.09 |  |
| Piemonte | 0.02 | 0.03 | 0.03 | 0.01 | 0.00 | 0.02 | 0.02 | 0.01 | 0.02 | 0.015 | 0.011 | 0.021 |  |
| Valle d'Aosta | 0.01 | 0.08 | -0.09 | -0.01 | 0.06 | -0.06 | 0.05 | 0.11 | -0.03 | 0.016 | 0.082 | -0.05 |  |
| Liguria | 0.04 | 0.05 | 0.03 | 0.03 | 0.05 | 0.02 | 0.04 | 0.05 | 0.03 | 0.037 | 0.048 | 0.027 |  |
| Lombardia | -0.02 | -0.01 | -0.01 | -0.03 | -0.03 | -0.02 | -0.05 | -0.05 | -0.03 | -0.036 | -0.032 | -0.023 |  |
| Trentino Alto Adige | -0.07 | -0.03 | -0.09 | -0.06 | -0.08 | -0.07 | -0.06 | -0.04 | -0.08 | -0.068 | -0.045 | -0.076 |  |
| Friuli-Venezia Giulia | 0.02 | 0.04 | 0.00 | 0.00 | 0.02 | -0.01 | -0.02 | 0.00 | -0.02 | 0.004 | 0.022 | -0.008 |  |
| Veneto | -0.05 | -0.03 | -0.05 | -0.04 | -0.02 | -0.04 | -0.05 | -0.05 | -0.04 | -0.044 | -0.034 | -0.043 |  |
| Emilia-Romagna | -0.02 | -0.02 | -0.01 | -0.03 | -0.05 | 0.00 | -0.03 | -0.04 | -0.01 | -0.022 | -0.032 | -0.01 |  |
| Toscana | -0.03 | -0.04 | -0.03 | -0.04 | -0.05 | -0.03 | -0.04 | -0.05 | -0.02 | -0.041 | -0.049 | -0.026 |  |
| Umbria | -0.03 | -0.04 | -0.03 | -0.03 | -0.04 | -0.04 | -0.06 | -0.07 | -0.05 | -0.046 | -0.053 | -0.034 |  |
| Lazio | 0.07 | 0.07 | 0.07 | 0.08 | 0.07 | 0.07 | 0.06 | 0.05 | 0.07 | 0.065 | 0.058 | 0.069 |  |
| Marche | -0.07 | -0.08 | -0.07 | -0.07 | -0.09 | -0.06 | -0.07 | -0.07 | -0.06 | -0.071 | -0.078 | -0.06 |  |
| Abruzzo | 0.05 | 0.08 | -0.02 | 0.09 | 0.07 | 0.03 | 0.10 | 0.14 | 0.07 | 0.071 | 0.097 | 0.044 |  |
| Molise | -0.01 | -0.02 | -0.02 | 0.00 | 0.01 | -0.04 | -0.02 | 0.00 | -0.04 | -0.005 | -0.001 | -0.029 |  |
| Campania | 0.17 | 0.16 | 0.16 | 0.17 | 0.16 | 0.17 | 0.17 | 0.17 | 0.17 | 0.168 | 0.163 | 0.162 |  |
| Puglia | -0.03 | -0.06 | 0.01 | -0.01 | -0.04 | 0.01 | 0.00 | -0.02 | 0.02 | -0.012 | -0.041 | 0.014 |  |
| Basilicata | -0.03 | -0.05 | -0.02 | -0.01 | -0.02 | -0.02 | 0.02 | 0.02 | 0.01 | -0.002 | -0.01 | -0.003 |  |
| Calabria | -0.01 | -0.04 | 0.01 | 0.02 | 0.01 | 0.02 | 0.03 | 0.02 | 0.02 | 0.014 | 0 | 0.017 |  |
| Sicilia | 0.09 | 0.05 | 0.12 | 0.11 | 0.08 | 0.13 | 0.12 | 0.09 | 0.14 | 0.104 | 0.072 | 0.126 |  |
| Sardegna | 0.00 | 0.02 | -0.03 | -0.01 | 0.02 | -0.03 | 0.00 | 0.02 | -0.03 | -0.004 | 0.018 | -0.03 |  |
|  |  |  |  |  |  |  |  |  |  |  |  |  |  |
| Winter |  |  |  |  |  |  |  |  |  |  |  |  |  |
| ITALY | 0.13 | 0.16 | 0.08 | 0.2 | 0.16 | 0.15 | 0.2 | 0.26 | 0.16 | 0.17 | 0.19 | 0.14 |  |
| Piemonte | 0.04 | 0.03 | 0.05 | 0.02 | 0.01 | 0.03 | 0.03 | 0.03 | 0.03 | 0.027 | 0.022 | 0.035 |  |
| Valle d'Aosta | -0.01 | 0.04 | -0.08 | -0.02 | 0.02 | -0.06 | 0.03 | 0.07 | -0.04 | 0 | 0.043 | -0.053 |  |
| Liguria | 0.05 | 0.06 | 0.05 | 0.04 | 0.05 | 0.02 | 0.04 | 0.05 | 0.03 | 0.044 | 0.055 | 0.033 |  |
| Lombardia | -0.01 | 0.00 | -0.01 | -0.02 | -0.02 | -0.01 | -0.04 | -0.04 | -0.03 | -0.03 | -0.026 | -0.019 |  |
| Trentino Alto Adige | -0.07 | -0.03 | -0.10 | -0.07 | -0.08 | -0.08 | -0.08 | -0.05 | -0.10 | -0.076 | -0.051 | -0.088 |  |
| Friuli-Venezia Giulia | 0.03 | 0.06 | 0.01 | 0.00 | 0.02 | -0.01 | -0.02 | 0.00 | -0.03 | 0.007 | 0.028 | -0.006 |  |
| Veneto | -0.05 | -0.02 | -0.05 | -0.04 | -0.02 | -0.04 | -0.05 | -0.05 | -0.04 | -0.044 | -0.033 | -0.046 |  |
| Emilia-Romagna | -0.02 | -0.02 | -0.01 | -0.03 | -0.05 | 0.00 | -0.03 | -0.04 | -0.01 | -0.026 | -0.037 | -0.012 |  |
| Toscana | -0.04 | -0.04 | -0.03 | -0.04 | -0.05 | -0.02 | -0.05 | -0.06 | -0.02 | -0.043 | -0.052 | -0.026 |  |
| Umbria | -0.03 | -0.04 | -0.03 | -0.04 | -0.04 | -0.04 | -0.07 | -0.08 | -0.06 | -0.049 | -0.056 | -0.037 |  |
| Lazio | 0.09 | 0.09 | 0.09 | 0.09 | 0.10 | 0.08 | 0.08 | 0.07 | 0.09 | 0.083 | 0.083 | 0.083 |  |
| Marche | -0.08 | -0.08 | -0.08 | -0.08 | -0.09 | -0.07 | -0.08 | -0.08 | -0.08 | -0.079 | -0.085 | -0.07 |  |
| Abruzzo | -0.03 | -0.02 | -0.03 | -0.01 | -0.02 | -0.02 | 0.00 | -0.01 | 0.00 | -0.015 | -0.014 | -0.02 |  |
| Molise | -0.01 | -0.02 | -0.02 | 0.00 | 0.02 | -0.05 | -0.01 | 0.02 | -0.04 | -0.004 | 0.006 | -0.035 |  |
| Campania | 0.19 | 0.19 | 0.18 | 0.24 | 0.23 | 0.23 | 0.24 | 0.24 | 0.23 | 0.221 | 0.221 | 0.213 |  |
| Puglia | -0.03 | -0.06 | 0.01 | -0.01 | -0.03 | 0.01 | 0.01 | -0.01 | 0.03 | -0.01 | -0.039 | 0.018 |  |
| Basilicata | -0.04 | -0.05 | -0.03 | 0.00 | -0.02 | -0.01 | 0.01 | 0.00 | 0.00 | -0.004 | -0.015 | -0.002 |  |
| Calabria | 0.01 | -0.03 | 0.03 | 0.03 | 0.02 | 0.02 | 0.04 | 0.03 | 0.04 | 0.026 | 0.009 | 0.031 |  |
| Sicilia | 0.12 | 0.07 | 0.16 | 0.14 | 0.10 | 0.17 | 0.15 | 0.12 | 0.18 | 0.137 | 0.097 | 0.171 |  |
| Sardegna | 0.02 | 0.04 | -0.03 | 0.00 | 0.02 | -0.02 | 0.01 | 0.04 | -0.03 | 0.004 | 0.031 | -0.03 |  |
|  |  |  |  |  |  |  |  |  |  |  |  |  |  |
| Spring |  |  |  |  |  |  |  |  |  |  |  |  |  |
| ITALY | 0.1 | 0.14 | 0.04 | 0.1 | 0.08 | 0.03 | 0.13 | 0.16 | 0.13 | 0.1 | 0.12 | 0.07 |  |
| Piemonte | 0.02 | 0.02 | 0.02 | 0.00 | 0.00 | 0.01 | 0.01 | 0.01 | 0.02 | 0.01 | 0.006 | 0.016 |  |
| Valle d'Aosta | 0.03 | 0.12 | -0.08 | 0.00 | 0.06 | -0.05 | 0.04 | 0.11 | -0.02 | 0.025 | 0.091 | -0.044 |  |
| Liguria | 0.04 | 0.05 | 0.04 | 0.04 | 0.05 | 0.03 | 0.03 | 0.04 | 0.03 | 0.039 | 0.048 | 0.032 |  |
| Lombardia | -0.02 | 0.00 | -0.01 | -0.03 | -0.03 | -0.02 | -0.04 | -0.05 | -0.03 | -0.033 | -0.03 | -0.021 |  |
| Trentino Alto Adige | -0.06 | -0.04 | -0.08 | -0.06 | -0.08 | -0.06 | -0.06 | -0.04 | -0.07 | -0.063 | -0.043 | -0.069 |  |
| Friuli-Venezia Giulia | 0.02 | 0.04 | 0.01 | 0.00 | 0.01 | -0.01 | -0.01 | 0.00 | -0.02 | 0.006 | 0.021 | -0.004 |  |
| Veneto | -0.05 | -0.02 | -0.05 | -0.04 | -0.02 | -0.04 | -0.04 | -0.04 | -0.03 | -0.041 | -0.032 | -0.04 |  |
| Emilia-Romagna | -0.01 | -0.02 | -0.01 | -0.02 | -0.04 | 0.00 | -0.02 | -0.04 | -0.01 | -0.019 | -0.029 | -0.008 |  |
| Toscana | -0.03 | -0.04 | -0.03 | -0.04 | -0.05 | -0.03 | -0.04 | -0.05 | -0.02 | -0.038 | -0.046 | -0.024 |  |
| Umbria | -0.04 | -0.04 | -0.03 | -0.03 | -0.03 | -0.04 | -0.05 | -0.06 | -0.04 | -0.043 | -0.048 | -0.033 |  |
| Lazio | 0.07 | 0.06 | 0.07 | 0.07 | 0.07 | 0.07 | 0.05 | 0.05 | 0.06 | 0.062 | 0.057 | 0.065 |  |
| Marche | -0.06 | -0.07 | -0.06 | -0.07 | -0.09 | -0.06 | -0.07 | -0.07 | -0.06 | -0.066 | -0.074 | -0.056 |  |
| Abruzzo | 0.00 | -0.01 | 0.00 | -0.01 | -0.02 | -0.01 | -0.01 | -0.01 | -0.01 | -0.01 | -0.013 | -0.011 |  |
| Molise | 0.00 | -0.01 | -0.02 | -0.01 | 0.00 | -0.04 | -0.02 | -0.01 | -0.04 | -0.008 | -0.004 | -0.029 |  |
| Campania | 0.16 | 0.14 | 0.17 | 0.16 | 0.15 | 0.16 | 0.17 | 0.16 | 0.17 | 0.161 | 0.151 | 0.163 |  |
| Puglia | -0.03 | -0.05 | 0.00 | -0.01 | -0.04 | 0.00 | 0.00 | -0.02 | 0.01 | -0.014 | -0.039 | 0.007 |  |
| Basilicata | -0.03 | -0.04 | -0.01 | 0.00 | 0.00 | -0.02 | 0.03 | 0.03 | 0.02 | 0.008 | 0 | 0.004 |  |
| Calabria | 0.00 | -0.03 | 0.01 | 0.03 | 0.01 | 0.03 | 0.03 | 0.02 | 0.03 | 0.018 | 0.002 | 0.023 |  |
| Sicilia | 0.09 | 0.04 | 0.11 | 0.11 | 0.08 | 0.12 | 0.13 | 0.10 | 0.15 | 0.103 | 0.074 | 0.123 |  |
| Sardegna | 0.01 | 0.02 | -0.02 | 0.01 | 0.04 | -0.02 | 0.01 | 0.03 | -0.02 | 0.005 | 0.026 | -0.022 |  |
|  |  |  |  |  |  |  |  |  |  |  |  |  |  |
| Summer |  |  |  |  |  |  |  |  |  |  |  |  |  |
| ITALY | 0.01 | 0.01 | 0.01 | 0.07 | 0.14 | -0.01 | 0.13 | 0.23 | 0.06 | 0.06 | 0.14 | 0.01 |  |
| Piemonte | 0.05 | 0.14 | -0.06 | 0.00 | -0.01 | 0.01 | 0.01 | 0.00 | 0.02 | 0.005 | 0.002 | 0.011 |  |
| Valle d'Aosta | 0.03 | 0.05 | 0.02 | 0.07 | 0.21 | -0.05 | 0.12 | 0.23 | 0.00 | 0.08 | 0.198 | -0.028 |  |
| Liguria | -0.03 | -0.01 | -0.02 | 0.03 | 0.04 | 0.02 | 0.03 | 0.04 | 0.03 | 0.03 | 0.043 | 0.02 |  |
| Lombardia | -0.05 | 0.00 | -0.08 | -0.03 | -0.03 | -0.02 | -0.04 | -0.04 | -0.02 | -0.033 | -0.031 | -0.022 |  |
| Trentino Alto Adige | 0.00 | 0.02 | -0.01 | -0.05 | -0.07 | -0.06 | -0.05 | -0.03 | -0.06 | -0.059 | -0.035 | -0.062 |  |
| Friuli-Venezia Giulia | -0.04 | -0.03 | -0.04 | 0.00 | 0.02 | -0.01 | -0.02 | -0.01 | -0.02 | -0.005 | 0.009 | -0.012 |  |
| Veneto | -0.01 | -0.01 | -0.01 | -0.03 | -0.02 | -0.03 | -0.04 | -0.04 | -0.03 | -0.035 | -0.03 | -0.032 |  |
| Emilia-Romagna | -0.03 | -0.03 | -0.02 | -0.02 | -0.04 | -0.01 | -0.02 | -0.03 | -0.01 | -0.019 | -0.025 | -0.01 |  |
| Toscana | -0.03 | -0.04 | -0.03 | -0.04 | -0.04 | -0.02 | -0.03 | -0.04 | -0.02 | -0.035 | -0.041 | -0.022 |  |
| Umbria | 0.05 | 0.04 | 0.06 | -0.03 | -0.04 | -0.03 | -0.05 | -0.06 | -0.04 | -0.04 | -0.046 | -0.027 |  |
| Lazio | -0.06 | -0.06 | -0.05 | 0.05 | 0.05 | 0.06 | 0.04 | 0.03 | 0.05 | 0.046 | 0.037 | 0.054 |  |
| Marche | 0.00 | 0.01 | -0.01 | -0.07 | -0.08 | -0.05 | -0.06 | -0.06 | -0.05 | -0.061 | -0.068 | -0.049 |  |
| Abruzzo | -0.01 | -0.03 | -0.01 | -0.01 | -0.01 | -0.01 | 0.00 | 0.00 | 0.01 | -0.001 | 0 | -0.007 |  |
| Molise | 0.11 | 0.11 | 0.10 | 0.03 | 0.04 | -0.02 | -0.01 | -0.01 | -0.03 | 0.002 | 0 | -0.014 |  |
| Campania | -0.01 | -0.04 | 0.02 | 0.10 | 0.10 | 0.10 | 0.10 | 0.09 | 0.10 | 0.102 | 0.099 | 0.096 |  |
| Puglia | -0.03 | -0.04 | -0.02 | 0.00 | -0.03 | 0.03 | 0.01 | -0.01 | 0.03 | -0.001 | -0.031 | 0.028 |  |
| Basilicata | 0.00 | -0.03 | 0.02 | -0.01 | -0.02 | -0.01 | 0.02 | 0.03 | 0.00 | -0.001 | -0.006 | -0.005 |  |
| Calabria | 0.08 | 0.04 | 0.10 | 0.02 | 0.01 | 0.01 | 0.03 | 0.02 | 0.02 | 0.014 | 0.001 | 0.017 |  |
| Sicilia | 0.00 | 0.01 | -0.03 | 0.07 | 0.04 | 0.09 | 0.09 | 0.07 | 0.11 | 0.078 | 0.05 | 0.1 |  |
| Sardegna | 0.01 | 0.01 | 0.01 | -0.02 | 0.02 | -0.03 | 0.00 | 0.02 | -0.02 | -0.006 | 0.015 | -0.027 |  |
|  |  |  |  |  |  |  |  |  |  |  |  |  |  |
| Autumn |  |  |  |  |  |  |  |  |  |  |  |  |  |
| ITALY | 0.01 | 0.05 | -0.02 | 0.08 | 0.05 | 0.03 | -0.02 | 0.01 | -0.02 | 0.01 | 0.02 | 0 |  |
| Piemonte | 0.03 | 0.04 | 0.02 | 0.02 | 0.01 | 0.02 | 0.02 | 0.01 | 0.02 | 0.018 | 0.016 | 0.021 |  |
| Valle d'Aosta | -0.02 | 0.04 | -0.08 | -0.02 | 0.01 | -0.05 | 0.02 | 0.04 | -0.02 | -0.008 | 0.025 | -0.041 |  |
| Liguria | 0.02 | 0.03 | 0.03 | 0.03 | 0.04 | 0.02 | 0.03 | 0.04 | 0.02 | 0.025 | 0.033 | 0.021 |  |
| Lombardia | -0.01 | 0.00 | 0.00 | -0.02 | -0.02 | 0.00 | -0.03 | -0.04 | -0.02 | -0.024 | -0.022 | -0.012 |  |
| Trentino Alto Adige | -0.05 | -0.02 | -0.06 | -0.05 | -0.06 | -0.05 | -0.05 | -0.03 | -0.07 | -0.052 | -0.033 | -0.056 |  |
| Friuli-Venezia Giulia | 0.02 | 0.04 | 0.01 | 0.02 | 0.04 | 0.00 | -0.01 | 0.01 | -0.01 | 0.009 | 0.027 | -0.002 |  |
| Veneto | -0.04 | -0.01 | -0.04 | -0.03 | -0.01 | -0.03 | -0.03 | -0.03 | -0.02 | -0.03 | -0.023 | -0.028 |  |
| Emilia-Romagna | -0.01 | -0.01 | 0.00 | -0.02 | -0.03 | 0.00 | -0.02 | -0.03 | 0.00 | -0.013 | -0.022 | -0.001 |  |
| Toscana | -0.02 | -0.03 | -0.01 | -0.03 | -0.04 | -0.02 | -0.03 | -0.04 | -0.01 | -0.029 | -0.037 | -0.016 |  |
| Umbria | -0.02 | -0.03 | -0.01 | -0.02 | -0.03 | -0.02 | -0.04 | -0.04 | -0.04 | -0.035 | -0.042 | -0.021 |  |
| Lazio | 0.07 | 0.07 | 0.06 | 0.07 | 0.06 | 0.07 | 0.04 | 0.03 | 0.05 | 0.053 | 0.047 | 0.056 |  |
| Marche | -0.05 | -0.06 | -0.04 | -0.05 | -0.07 | -0.04 | -0.05 | -0.06 | -0.05 | -0.05 | -0.059 | -0.039 |  |
| Abruzzo | -0.02 | -0.02 | -0.02 | -0.01 | -0.01 | -0.01 | -0.01 | 0.00 | -0.01 | -0.011 | -0.011 | -0.013 |  |
| Molise | 0.00 | -0.01 | -0.01 | -0.01 | 0.00 | -0.04 | -0.01 | 0.00 | -0.03 | -0.005 | -0.001 | -0.022 |  |
| Campania | 0.14 | 0.14 | 0.12 | 0.13 | 0.12 | 0.13 | 0.11 | 0.11 | 0.10 | 0.122 | 0.125 | 0.108 |  |
| Puglia | -0.02 | -0.05 | 0.00 | -0.01 | -0.03 | 0.00 | -0.01 | -0.02 | 0.01 | -0.012 | -0.032 | 0.006 |  |
| Basilicata | -0.03 | -0.04 | -0.01 | -0.01 | -0.01 | -0.01 | 0.01 | 0.01 | 0.01 | -0.002 | -0.009 | -0.001 |  |
| Calabria | -0.02 | -0.04 | -0.01 | 0.02 | 0.01 | 0.01 | 0.01 | 0.01 | 0.00 | 0.002 | -0.005 | 0.001 |  |
| Sicilia | 0.05 | 0.02 | 0.06 | 0.09 | 0.07 | 0.07 | 0.06 | 0.05 | 0.06 | 0.057 | 0.045 | 0.059 |  |
| Sardegna | -0.01 | 0.00 | -0.02 | -0.01 | 0.00 | -0.02 | -0.01 | 0.01 | -0.02 | -0.01 | 0.003 | -0.023 |  |

*
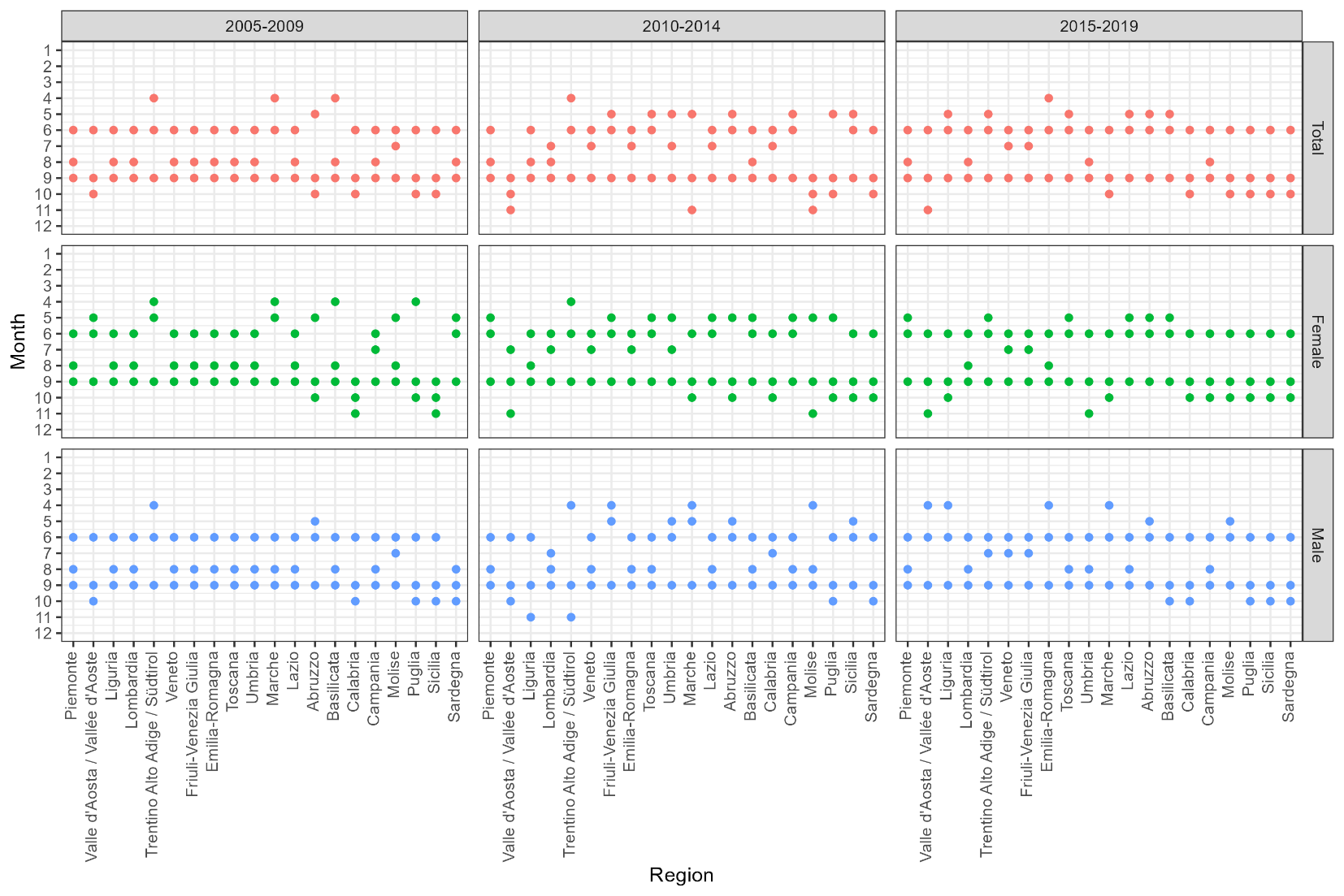
Figure S10. Months used for the construction of the baseline mortality level, by region, year and sex, 2005-2009, 2010-2014, 2015-2019*
